# A pan-European art trade in the late middle ages: Isotopic evidence on the master of Rimini enigma

**DOI:** 10.1371/journal.pone.0265242

**Published:** 2022-04-12

**Authors:** Wolfram Kloppmann, Lise Leroux, Philippe Bromblet, Pierre-Yves Le Pogam, Anne Thérèse Montech, Catherine Guerrot

**Affiliations:** 1 BRGM, French Geological Survey, Orléans, France; 2 LRMH, Champs-sur-Marne, France; 3 CRC-USR3224, MNHN-CNRS-MCC, Paris, France; 4 CICRP Belle-de-Mai, Marseille, France; 5 Département des Sculptures, Musée du Louvre, Paris Cedex, France; Università degli Studi della Campania “Luigi Vanvitelli, ITALY

## Abstract

The identity of artists and localisation of workshops are rarely known with certainty before the mid-15^th^ century. We investigated the material used by one of the most prolific and enigmatic medieval sculptors, the Master of the Rimini Altarpiece or Master of Rimini, active around 1420–40. The isotope fingerprints (Sr, S and O) of a representative corpus of masterpieces but also minor artworks, attributed to the Master of Rimini and his workshop, are virtually identical, demonstrating the unity of the corpus and a material evidence behind the stylistic and iconographic ascriptions. The material used is exclusively Franconian (N-Bavarian) alabaster, 600 km distant from the supposed zone of activity of the Master of Rimini workshop according to recent literature. The same material was later used by the prominent Late Medieval German carver Tilman Riemenschneider, active in Würzburg after 1483, whose small corpus of alabaster sculptures we have been able to characterize almost entirely. Based on these findings, we propose here an alternative to the prevailing hypothesis of a Flemish or N-French workshop being founded on similarities of the Rimini sculpture with motives in Flemish and French painting. Our scenario, returning to the initial proposal of a German localisation of the Master of Rimini workshop, assumes the migration of an artist, perhaps trained in the Low Countries or strongly inspired by the Flemish art, to Southern Germany where he founded a highly productive export workshop, well situated on the crossroads of medieval trade, with a pan-European radiance. This study sheds a spotlight on the on the trade networks of luxury goods, the raw material used for their production, and the high-end art market in Europe as well as on international migration of artists and styles, at the eve of the Renaissance.

## Introduction

The identity and location of the workshop of the enigmatic Master of the Rimini Altarpiece, active around 1420–40, remain unknown, in spite of an abundant production and Europe-wide exportation [1, 2] of his sculptures, exclusively cut in alabaster.

**Fig 1:**
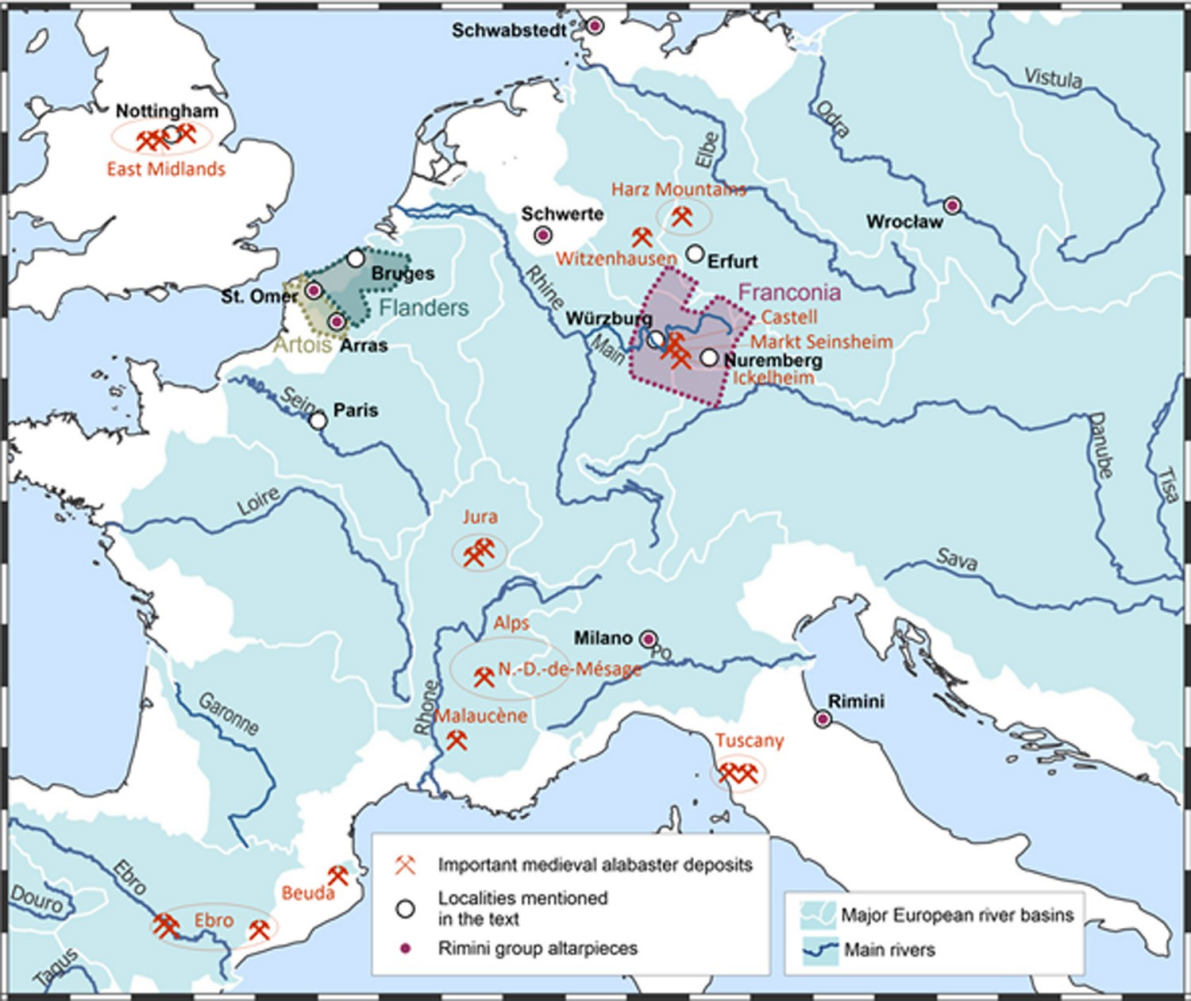
Potential localisations and export destinations of the Master of Rimini workshop. Major historical alabaster-producing regions in late medieval W Europe and newly investigated deposits in Franconia; historical localisation of the main known sculptural ensembles related to the Rimini workshop (violet points, Arras: attribution uncertain); localities mentioned in the text; potential areas of activity of the Master of Rimini workshop (historical provinces of Franconia in violet, Flanders in green, and Artois in beige, in their 15^th^-early 16^th^ cent. limits). Background map: Major European river basins in light blue, rivers in blue. The map was created using open source Q-GIS 3.10 software, underlying data (borders, hydrography) are from the free Esri Geoportal Server (https://www.esri.com/en-us/arcgis/products/geoportal-server/overview).

The masterpiece of the “exceptionally skilled” [1] sculptor, commonly referred to as the Master of the Rimini Altarpiece or Master of Rimini, is a crucifixion group including twelve apostles, originally placed in the church of Santa Maria delle Grazie, near Rimini, Italy. It was acquired by the Liebieghaus Museum (Frankfurt, Germany) in 1913 [3, 4]. Distinctive stylistic features of this group led to numerous attributions of alabaster artwork, spread all over Europe, to his workshop. The quality of the Rimini altarpiece and several other stylistically and materially closely related sculptures reached an artistic level designating them to the high-end art market in the early 15^th^ century. The few known or supposed clients are princely families like the Italian Borromei and Malatesta [1] and rich abbeys, from Arras (N France, Fig 1) to Wrocław (Poland). However, what Kim Woods specified as “Rimini group” of artworks [1] encompasses a large range of artistic qualities. At the lower end of this spectrum, numerous minor works exist, showing similarities with the Rimini Altarpiece and other masterpieces attributed to the Master of Rimini. They are considered as workshop productions that may have been destined to the anonymous art market and to personal devotion.

The “Master of Rimini” remains mysterious in several respects: Can the artwork from the “Rimini group” be attributed to the same hand, the same workshop or even a group of workshops and stylistic followers? Can the Master be qualified as itinerant or migratory artist with or without a stable workshop? If a stable workshop existed, where was the production located and where was the nodal point of this pan-European trade network?.

Only a rough estimate of the surviving work is possible due to the diversity within this group hindering a clear definition of the limits between the production of the Master of Rimini or his workshop and possible followers [5]. The broad diffusion of artwork linked to the Master of Rimini with known provenance (Fig 1) in Europe makes a geographic localisation of the workshop impossible. It is again on a stylistic basis that the Master of Rimini was first qualified as typically Middle Rhenish, south of Cologne, notably by Swarzenski [3] who purchased the Rimini group for Frankfurt and initiated the scientific research on medieval alabaster sculpture outside England. Swarzenski formally discards from the very beginning the possibility that the Master of Rimini was Italian [6] already mentions a second hypothesis of a Netherlandish-French origin of the sculpture of the Rimini group, first formulated by Volbach [7], that is now prevailing, even though, as Kim Woods states in her landmark monography on medieval alabaster sculpture, the possibility that the Master of Rimini was German has never been dismissed [2, 5, 8]. Arguments of the proponents of a Flemish origin [1, 2, 4, 9–12] are, on the one hand, the iconographic proximity to paintings by Rogier van der Weyden and the Master of Flémalle and to the painted representation of monochromatic sculptures and other elements of Jan van Eyck’s Gent altarpiece [1, 2, 4], and, on the other hand, the fact that the Netherlands, much like Burgundy and England, were exporting gothic art at large scale [7]. Recently, parallels with a 14^th^ century French silk painting have been pointed out [13]. Undeniably, the Rimini style is multifaceted and other artistic influences, notably from Central Europe have been discerned [8, 14].

Our study sheds a new light on the Rimini enigma, from an angle so far underexploited in a research dominated by art historical, stylistic and iconographic approaches. It focuses on the material used for the artworks attributed to the Rimini group. Historically, varieties of both calcite and gypsum/anhydrite, and, in some cases, onyx, i.e. a banded form of the silicate mineral chalcedony, were designated as "alabaster." The name stems from the calcite variety, known as "Egyptian" or "Oriental" alabaster. The stone of Egyptian *alabastra*, small-sized ointment vessels, is a brownish, banded calcium carbonate (CaCO_3_) and geologically speaking a calcareous sinter or travertine [15]. In our study, we use the term for what Aston et al. referred to as”real” alabaster [15], which alone in medieval Europe was widespread and used for sculpture, the fine-grained to cryptocrystalline variety of gypsum, a hydrous calcium sulfate (CaSO_4_·2H_2_O) or its anhydrous variety the anhydrite (CaSO_4_) [16, 17].

**Fig 2:**
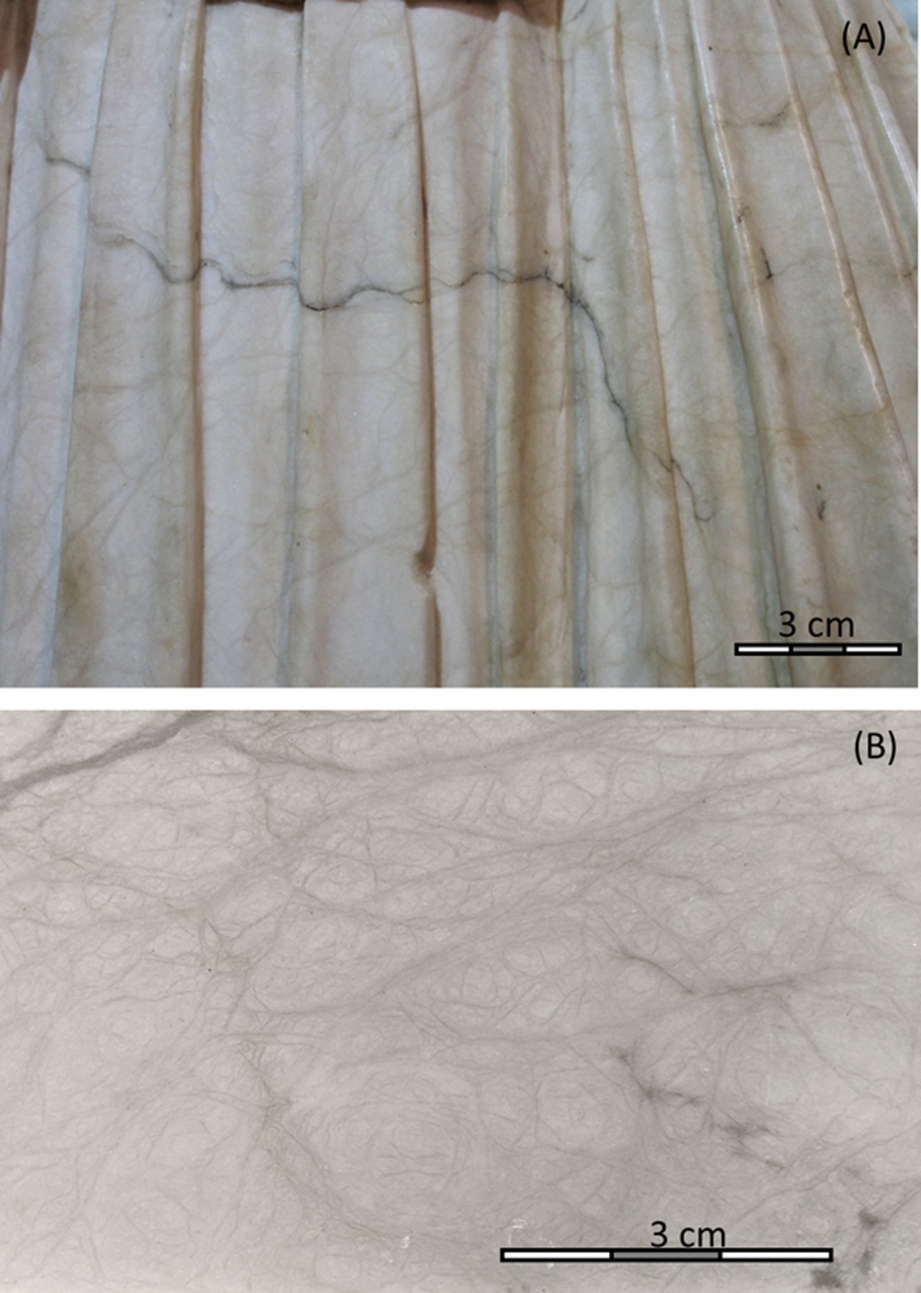
Characteristic clear grey and dark veins of the alabaster used by the Rimini workshop. **(A)** Back side of the Swooning Mary group of the Rimini Crucifixion, Liebieghaus Skulpturensammlung **(B)** polished surface of the Ickelheim alabaster.

It was Kim Woods who first stated similarities in the appearance (“similar, albeit not identical” [1]) of the alabaster used both by the Master of Rimini and another sculptor and wood carver, Tilman Riemenschneider (1450–1531). He is among the most prominent names of late medieval sculpture and his biography and artistic production, half a century after the obscure Master of Rimini, are documented in detail [8, 18]. Both used an alabaster described as “covered with distinctive network of grey veins around 2 mm thick and resembling blood vessels” [1] (Fig 2A). We make use of the proven capacity of Sr, S and O isotope fingerprints to discriminate historical European alabaster deposits [19–21] to verify this intriguing resemblance, to test the provenance of the material used by both the Master of Rimini and by Riemenschneider, and, ultimately, to provide new evidence to the identity of the Master of Rimini and the location of his workshop by identifying his supply chains. We then confront our geochemical results with the rare written medieval sources on artwork of the Rimini group that we have investigated in some more detail, going back to some of the original manuscripts.

**Fig 3:**
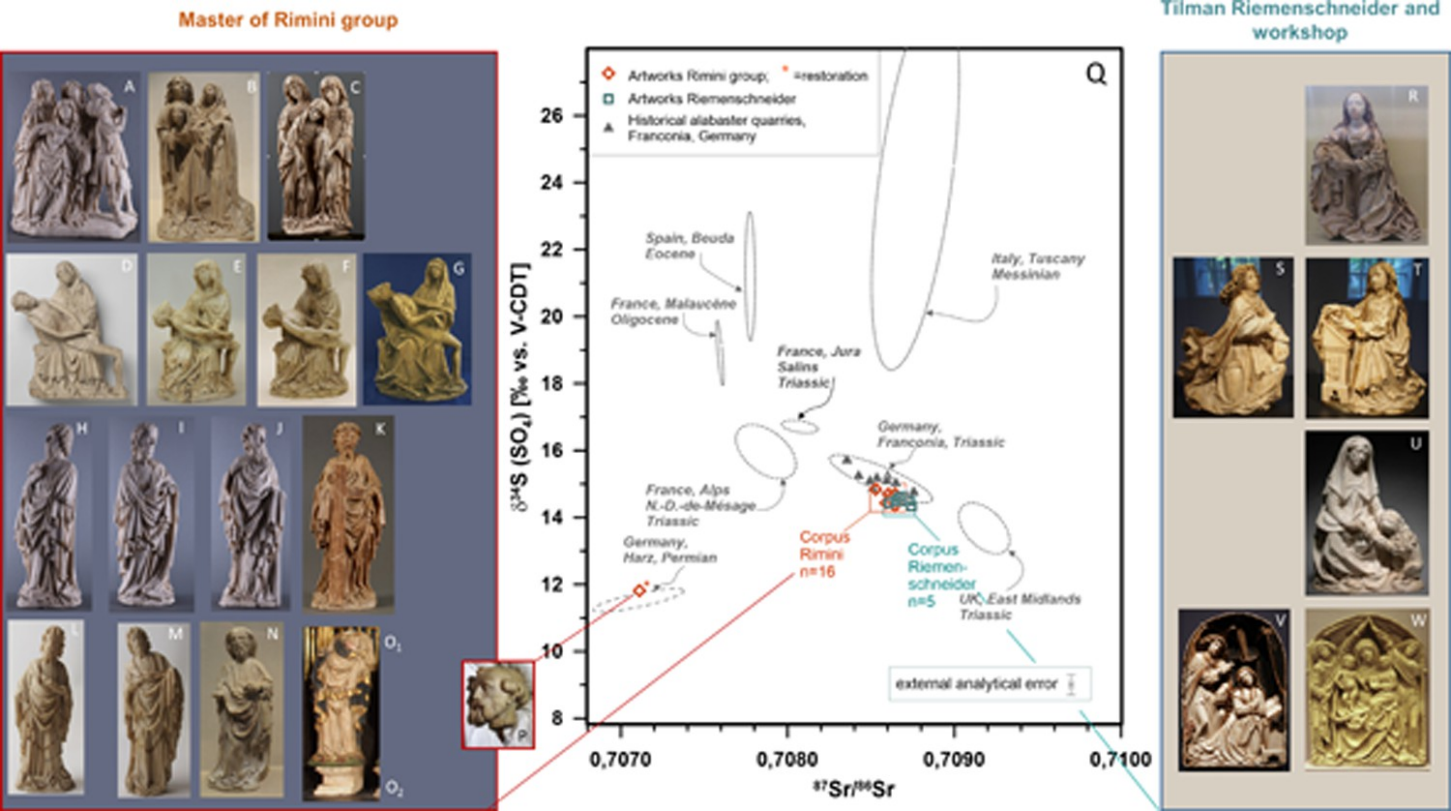
A-M: Master of Rimini group. **A-C**: Swooning Mary groups (A: Liebieghaus Museum, Rimini altarpiece, B: Louvre Museum, C: National Museum of Warsaw); **D-G**: Pietàs (D: Rijksmuseum, Amsterdam, E: Mittelrheinmuseum Koblenz, F: Museum am Dom, Würzburg, G: Deutschordensmuseum, Bad Mergentheim); **H-O**: Apostles (H-J: Rimini Altarpiece, Liebieghaus Museum, Frankfurt, K: J. Paul Getty Museum, L-M: Musée de l’Hôtel Sandelin, N: Louvre Museum, O_1_-O_2_: Schwerte, Saint Viktor church); **P**: Replacement of the head of Saint Peter, Liebieghaus Museum, Rimini altarpiece. **Q: δ**^**34**^**S *vs*.** ^**87**^**Sr/**^**86**^**Sr for artwork and Franconian quarries.** For comparison: principal deposits previously identified to have delivered alabaster for 14^th^ to 16^th^ century sculpture in W Europe [19–21], renormalised to V-CDT. **R-W: Riemenschneider and workshop**. **R-T**: Annunciation groups (R: Louvre Museum, S-T: Rijksmuseum), **U**: Saint Jerome, Cleveland Museum of Fine Art, **V**: Annunciation, D. Katz Gallery, **W**: Madonna and the Child in the lap of Saint Anne, Museum für Franken. **Photographic credits:** National Museum in Warsaw **(C)**, reprinted from https://cyfrowe.mnw.art.pl/en/catalog/616803 under a CC BY license, Liebieghaus Museum **(A, H, I, J)**, kindly provided by Liebieghaus Museum with permission to publish under a CC BY license, Rijksmuseum **(D)**, reprinted from https://www.rijksmuseum.nl/en/collection/BK-NM-11667 under a CC BY license, J. Paul Getty Museum **(K)**, reprinted from http://www.getty.edu/art/collection/objects/284883/master-of-the-rimini-altar under a CC BY license, Cleveland Museum of Fine Art **(U)**, reprinted from https://www.clevelandart.org/art/1946.82 under a CC BY license, D. Katz Gallery **(V)** kindly provided by D. Katz Gallery with permission to publish under a CC BY license. Other photos **(B, E-G, L-P, R-T, W)** author’s own photographic work (Wolfram Kloppmann).

Two late medieval sources exist that reveal a Europe-wide trade of alabaster sculpture in the first half of the 15^th^ century, the time the Rimini-workshop was active. The first concerns a group representing the swooning Mary, the so-called “Three Maries”, now in the National Museum of Warsaw and investigated in our study (Fig 3C). It was part of a Crucifixion, now attributed to the Master of Rimini, acquired in 1431 for the church of our Lady of the Sand in Wrocław (Poland) by the Abbot Jodocus of the Wrocław Augustinian friary from a Parisian merchant [3, 10, 11, 22, 23], even if Woods [1] points out that the term for designing Paris in the Wrocław trade record (*parysiis in montanis*) is ambiguous. We resolve this ambiguity by identifying the expression “*in montanis”* as a later transcription error that should read “*cum montanis*” as in the earliest preserved copy of Jodocus’ lost manuscript, pointing to a Calvary-like arrangement (see detailed discussion on this point in the S1 Appendix and S1, S2 Figs in S1 File).

The second documented acquisition, one year later, in 1432, was made by Jean de Clercq, the Abbot of the Saint Vaast Abbey at Arras (N-France, Fig 1), who bought an alabaster Coronation of the Virgin group including the twelve apostles, from a German merchant [24–26]. This ensemble is lost but alabaster artwork from the same period is still conserved in the same ancient Artois region, notably the four apostles (Fig 3L and 3M), supposedly part of a similar altarpiece with twelve apostles, acquired in 1429 by the canon Gauthier Ponche for the Saint-Omer Cathedral [24], now in the Musée de l’Hôtel Sandelin in Saint-Omer, that can be counted in the group of Rimini art [24]. Both written sources have been used as arguments for situating the workshop in Paris [4, 13], N-France or S-Netherlands [1] or in Germany [27, 28].

Whereas the Master of Rimini and his workshop exclusively used alabaster, this material is an exception in the works of Tilman Riemenschneider, one of the most accomplished artists of Late Gothic sculpture. After uncertain years of apprenticeship and journeying, he settled down definitely in Würzburg (Southern Germany) in 1483, where he worked in lime-wood, sandstone, and limestone [18]. Until recently, only five alabaster sculptures were attributed to Riemenschneider and his workshop [29], a Saint Jerome, now in The Cleveland Museum of Art, the Virgin of the Annunciation held by the Louvre (Paris), a complete Annunciation group, Virgin and Angel Gabriel, at the Rijksmuseum (Amsterdam), and a Saint Barbara, now in a private collection in Bremen. Two bas-reliefs were newly added to Riemenschneider’s alabaster corpus, a Madonna and the Child in the lap of Saint Anne (a so-called Anna Selbdritt group), now in the Museum für Franken (Würzburg) [30] and an Annunciation, formerly in a private collection in Munich and exposed in the Munich National Museum, currently for sale [31]. Reputedly, altars in the chantry of the Würzburg Cathedral were decorated with alabaster statues by Riemenschneider’s hand, now lost [29]. The Cleveland Saint Jerome and the Louvre Virgin can be traced back to the collection of a clergyman in Erfurt [32], in the 19^th^ century and are supposed to have been commanded by the clergy of Erfurt for the church of Saint Peter abbey. They were sold after 1892 from a private collection in Dieburg near Frankfurt [33] and their attribution to Riemenschneider dates back to 1906 [34] for the Louvre Virgin and to 1909 [33] for the Cleveland Saint Jerome.

Riemenschneider’s infrequent alabaster works are rather largely spread in time [8]: they cover a period from 1485–1487 (Amsterdam Annunciation group) to 1505–10 (Saint Jerome) [29, 35], all dating being based on stylistic comparisons. The reasons for using alabaster intermittently can only be suspected. The commands from Erfurt might have explicitly specified and even furnished the material to be used [29]. Indeed, Permian alabaster was quarried north of Erfurt in the South Harz region, notably in Nordhausen (Thuringia) but first written records on this deposit are from the mid-16^th^ century [36], and northwest of Erfurt in Witzenhausen (Hesse), exploited as early as 1458 [10]. Riemenschneider may have known alabaster from his youth in the Harz region [18], as supposed by Justus Bier [29] but also discovered it in Southern Germany where it abounds in the environs of his home town Würzburg.

In this study we report isotope analyses of a near complete corpus of the surviving Riemenschneider alabaster sculptures (six out of seven) and a representative selection of sculptures attributed to the Rimini group, including masterpieces like the Rimini altar and the Wrocław group but also smaller individual works with undeniable serial production. Our data base on European alabaster [19, 20] is completed by new data on historical quarries south German, Franconian alabaster, one of the two possible sources for the Riemenschneider workshop.

## Materials and methods

### Artwork included in this study

We have obtained and analysed samples from fifteen artworks, now scattered worldwide, attributed to the “Rimini group” [1], to the Master’s own hand, his workshop and hypothetical followers,. Our corpus includes four statues and groups from his most prominent opus, the Rimini altarpiece, recently restored at the Liebieghaus Museum (Frankfurt, Germany), plus one later replacement. The other works are an Apostle (RF 4402) and the Swoon of the Virgin (RF 1639) from the Louvre Museum (Paris, France), the Saint Philip (2015.58) of the J. Paul Getty Museum (Los Angeles, USA), the Swoon of the Virgin (the so-called “Three Maries” group, Śr.402) of the National Museum of Warsaw (Poland), the Pietà (BK11667) of the Rijksmuseum (Amsterdam, Netherlands), the Pietà (P 1990/13) of the Mittelrheinmuseum (Koblenz, Germany), the Pietà (Inv. 491) of the Museum am Dom (Würzburg, Germany), and the Pietà of the Deutschordensmuseum (Bad Mergentheim, Germany). Two of the four Apostles (2911.3 and 2911.4) of the Musée de l’Hôtel Sandelin (Saint-Omer, France) were investigated, as well as one Apostle and his pedestal in the Saint Viktor church in Schwerte (Germany), where eight alabaster apostles and a Christ in Majesty were integrated in a later wooden Antwerp altarpiece.

Of the seven known alabaster works attributed to Riemenschneider and his workshop, we have been able to characterize six, including the Saint Jerome (CMA 1946.82), held by the Cleveland Museum of Fine Art (Ohio, USA), the Virgin of the Annunciation (RF 1384) at the Louvre, the Annunciation Virgin and Angel (BK-16986-A and B) at the Rijksmuseum, the Anna Selbdritt (ZV67983) of the Museum für Franken (Würzburg, Germany), and the Annunciation currently at the Daniel Katz Gallery (London, UK).

For all museum/collection samples, the inventory numbers of the sampled artwork, names and locations of museums are reported in S1 Table in S1 File “Analyzed artwork”, column “Current situation” Table, images in Fig 3. All necessary permits were obtained for the described study, which complied with all relevant regulations.

### Identification of historical quarries in Southern Germany

The hypothesis of a South German or Central German origin for the alabaster used by Riemenschneider directed our research to the Würzburg region (Franconia, N-Bavaria, Germany). Geologically, all Franconian alabaster belongs to the Upper Triassic Ladinian to Karnian (Keuper) evaporites [37] that regionally comprise two gypsum complexes intercalated in grey, green and dark red marls: a massive white gypsum bank of around 10 m (the so-called “Grundgips”) and a less continuous level of gypsum nodules, attaining diameters up to 1.1 m, locally forming “nests”. This higher level has delivered alabaster, frequently containing argillaceous flasers and veins and rarely pure white whereas some coloured varieties were sought for [37].

The gypsum industry was and is still active in this region but the historical alabaster quarries in Southern Germany (Franconia) are scarcely documented in recent literature [37]. Fortunately we dispose of two 18^th^ century written sources describing in detail the ancient alabaster exploitations of the municipalities of Castell [38] and of Ickelheim [39], respectively 35 and 65 km SE from Würzburg (Fig 1). The Castell alabaster with a grey, a white and a reddish variety was used at least from the 16^th^ century onwards. The first text source on its use [38] dates back to 1578 and it was widely employed in the 16^th^ to 18^th^ centuries for decorative elements in religious architecture, mainly the grey variety due to its resemblance to marble. Following the anonymous description [38] of 1791, we localized the historical exploitations accurately to a few 100 m and analysed three samples of massive and nodular alabaster.

The Ickelheim deposit is described in great detail by Hofmann in 1757. He states the opening, of a “new quarry”, in 1748, whereas the nearby “old quarry” had reputedly been exploited for “400 years”. He also mentions the exploitation of alabaster nodules from the vineyards in the vicinity, with frequent large scale transports to Nuremberg. His description of the material is most intriguing: the colour is “throughout white” with “subtle veins of the same white colour” whereas some nodules contain black veins. This corresponds precisely to Kim Woods’ macroscopic description of the material used by the Master of Rimini [1] (Fig 3). The dimensions he provides for the “stones” or nodules are mostly 30x60 cm and the heaviest may attain 200–300 kg so that, for funeral effigies, “four of them need to be skilfully assembled”. We indeed state that neither of the individual statues in our corpus exceeds 60 cm. One exceptional case of a bigger sculpture associated to the Rimini workshop, the Pietà [27] of the Louvre Museum (R.F. 1807, H: 0.95, W: 0.83 m., D: 0,38 m), not included in this study due to its fragility, is distinctly composite and not monolithic. For some sculptures related to the Rimini group, it has been supposed that their geometry was determined by the dimensions and rounding of the used alabaster piece, notably for the Mergentheim Pietà [40] (Fig 3G) so that it is likely that indeed nodules and not banked alabaster levels were used.

Hofmann’s topographic description is sufficiently precise to locate with a high degree of confidence the historical locations of the “new” and also of the “old” quarry even if the alabaster hosting marls are rapidly eroded. The Ickelheim vineyards are still delivering alabaster nodules of decimetric size. On total, we analysed four nodules and fragments from four different locations SE of the village.

The third sampled Franconian deposit of similar geological age (three samples), Markt Seinsheim, is mentioned in 1840 to have delivered alabaster and gypsum [41]. We sampled white gypsum from abandoned exploitations at two different locations in distinct stratigraphic positions: the Karnian massive stratiform “Grundgips” layer, and the Ladinian to Karnian massive to nodular alabaster contained in the marls of the Estheria beds.

Details are provided in S2 Table in S1 File.

### Sampling

The required minimum quantity for a complete isotope analysis (Sr, S, and O isotopes) using the method described in Kloppmann, Leroux (19) is less than 20 mg. This corresponds to a tiny flake of around 2 x 2 x 2 mm. Flakes, sampled with a miniature chisel on a non-carved, non-visible surface of the sculpture (e.g. rear surface or base) were preferred to micro-drilling for two reasons: (1) It is possible to detect and correct any treatment or contamination of the surface by manual cleaning under a microscope, (2) there is less aesthetic impact as the non-carved surfaces frequently have defects such as drilled fixing holes or irregular surfaces allowing discrete sampling to be undertaken. We strictly avoided any suspected or visible repairs or fixings where gypsum plaster/mortar were present as these are highly contaminant for our method. We also avoided or cleaned mechanically, whenever possible, under a binocular microscope, by scraping with pointed tweezers, any surface treatments (patina, wax, whitewash,…) to obtain unaltered isotope signatures of fresh material.

### Analyses

The samples are crushed, weighed and slowly dissolved in a closed tube filled with 50 ml of Millipore® distilled water at 50°C in an oven for at least one week. After filtration, the 50 ml solution is divided in three aliquots; two aliquots of 5 ml are used for Sr isotopes and elemental analysis, the remaining 40 ml for sulphates isotopes. Sulphates are precipitated as BaSO_4_ from the filtered solution by adding acidified BaCl_2_ solution (5% HCl). The precipitate is then filtered off and left to dry and a fraction (≈350 μg) of BaSO_4_ is mixed with vanadium pentoxide in a tin capsule [42], injected in a flash combustion elemental analyser (Flash EA) where BaSO_4_ is reduced to SO2 at 1700–1800°C. The purified SO_2_ is analysed for S isotopes by a continuous flow isotope ratio mass spectrometer (CF-IRMS: Thermo Delta Plus XP). An aliquot of the BaSO_4_ (≈200μg) is placed in a silver capsule, injected in a high temperature conversion elemental analyser (TC/EA) reactor with a graphite insert at 1450°C. The resulting CO is analysed by CF-IRMS for oxygen isotopes. The isotopic composition of sulphur is expressed in the usual delta notation as a per mil (‰) deviation of the heavy-to-light isotope abundance ratio (^34^S/^32^S, ^18^O/^16^O) in the sample, with respect to international standards [43]. ^34^S/^32^S, including previously reported values for the historical quarries [19, 20] have been (re-)normalised to the V-CDT standard using the following most recent δ^34^S values (‰ *vs*. V-CDT) for the BaSO_4_ sulphate reference materials provided by the IAEA and the NBS: IAEA-SO-6 (-34.05‰), IAEA-SO-5 (0.49‰), NBS127 (21.12‰).

Oxygen isotopes are reported as δ^18^O with respect to the V-SMOW standard. Sulphur and oxygen isotopes are measured twice. The error, based on repeated measurements of international and in-house standards, is 0.5‰ for δ^18^O and 0.3‰ for δ^34^S (1σ following common practice for IRMS [44]).

Chemical purification of Sr is performed using an ion-exchange column (Sr-Spec) before mass analysis according to a method adapted from Pin and Bassin [45], with total blank <1 ng for the entire chemical procedure. After chemical separation, around 150 ng of Sr is loaded onto a tungsten filament with a tantalum activator and analysed with a Finnigan MAT262 multi-collector thermal ionization mass spectrometer (TIMS). The measured ^87^Sr/^86^Sr ratios are normalized to a ^86^Sr/^88^Sr of 0.1194. An average internal precision of ± 10 x 10^−6^ (2σ_m_) was currently obtained during this study. The reproducibility of the ^87^Sr/^86^Sr ratio measurements was tested through repeated analyses of the NBS 987 standard for which we obtained a measured mean value of 0.710245 ± 11 x 10^−6^ (2σ; n = 324) during the period of analysis. Sample ratios were normalized to the certified value of the NBS987 (0.710240).

## Results

### Isotope fingerprinting

All samples were analysed for their isotopic composition of strontium, sulphur and oxygen, following the protocol described in the Materials and Methods section. Results are provided in S3 Table in S1 File, Fig 3, and S4 Fig in S1 File.

All sculptures of our Rimini corpus show very homogeneous strontium and sulphur isotope signatures compared to the overall variability of the alabaster deposits [19] with mean values and standard deviations of respectively 0.70862 ± 0.00003 (or ±0.00006, 2σ) (n = 16) for ^87^Sr/^86^Sr compared to an analytical uncertainty around 0.000007, and of 14.6 ± 0.1 ‰ *vs*. V-CDT (n = 16) for δ^34^S. For δ^34^S, the standard deviations are smaller than the analytical uncertainty of 0.3 ‰. Oxygen isotope values are more variable with a mean δ^18^O of 12.8 ± 0.8 ‰ *vs*. V-SMOW (n = 16) compared to an analytical uncertainty of 0.5 ‰. The head of Saint Peter in the Rimini Altarpiece now in the Liebieghaus (INV 418), considered as a later addition, clearly represents an outlier, with a ^87^Sr/^86^Sr of 0.707111 ± 0.000008 and a δ^34^S of 11.8 ± 0.3 ‰ *vs*. V-CDT (Fig 3Q).

The values of the artworks attributed to Riemenschneider and workshop fall in the same field as the Rimini sculptures (Fig 3R, S4 Fig in S1 File.) with mean values, respectively for ^87^Sr/^86^Sr, δ^34^S, and δ^18^O, of 0.70868 ± 0.00005 (n = 6), 14.5 ± 0.1 ‰ *vs*. V-CDT (n = 5), and 12.8 ± 0.9 *vs*. V-SMOW.

The Franconian quarries in the Steigerwald region SE of Würzburg show a mean ^87^Sr/^86^Sr of 0.70856 ± 0.00012 (n = 10). The mean δ^34^S value is 15.1 ± 0.3 ‰ *vs*. CDT (n = 10) and the mean δ^18^O is 13.5 ± 0.4 ‰ *vs*. V-SMOW (n = 10). For the both latter elements, the standard deviation of the raw materials is smaller than the analytical uncertainty. No distinction of the individual quarries (Ickelheim, Castell, Markt Seinsheim) is possible in this group based on isotopic compositions.

## Discussion

The most striking fact of our results is the isotopic homogeneity of the artworks attributed to the Master of the Rimini Altarpiece and workshop, compared to the overall variability of the principal European alabaster deposits [19] (Fig 3Q). This is particularly true for the Sr and S fingerprints whereas the oxygen signatures are more variable (S4 Fig in S1 File.), which is usually the case for the gypsum and anhydrite so far investigated [19]. We can conclude that the Rimini workshop used a single source of alabaster supply, likely a single quarry, which is compatible with the visual homogeneity of the material stated before [1]. This fact is most astonishing in the context of the current hypothesis of the workshop being situated in or around Flanders. The Low Countries have indeed a strong tradition of alabaster carving but no local alabaster sources. Given the large variety of provenances of imported material available to a supposedly Flemish workshop, the very selective supply would indicate either an aesthetic choice or a strong traditional, commercial or personal link with a particular alabaster-producing region. The homogeneous isotope signatures also corroborate the stylistic attribution of all investigated alabaster sculptures to a common workshop or group of workshops, indicating that the “Rimini-style” is indeed recognizable among those of the early 15^th^ century, albeit the material may have played some role in the attributions. This homogeneity concerns both the masterpieces attributed to the hand of the Master of Rimini himself and minor pieces like the Pietà groups (Fig 3D–3G) that have been supposed to be, at least partly, serial workshop productions [4]. Particularly instructive examples in this respect are the Pietà groups conserved in Koblenz (Fig 3E) and Würzburg (Fig 3F) virtually identical in their dimensions and details. Material homogeneity indicates a strong link between the workshop leader and apprentices and maybe also possible imitators or followers. If the latter existed, they used the same material which, again, indicates either a close aesthetic association of the sought style with a specific type of alabaster or a local/regional supply for the main workshop and possible satellite workshops. Similarly, the Riemenschneider workshop used a single alabaster source, in spite of the only occasional employ of this material and the large temporal spreading of the concerned artworks.

None of the historical European deposits identified previously [19], corresponds to the observed isotope signatures of the two artwork corpuses. The fact that the geochemical signatures of the productions of the Rimini and Riemenschneider workshops are identical for the investigated corpuses, points to a South-German supply for both.

Concerning the two hypotheses prevailing so far on the scarce alabaster sculptures by Riemenschneider and his workshop [1], we can rule out with certainty that the historical material originates from the Permian gypsum and anhydrite deposits of the Harz mountains, their very distinctive fingerprints being incompatible with those of the artworks. The remaining hypothesis for the original material is that of a local supply around the city of Würzburg where Riemenschneider resided for nearly five decades. Indeed, we state that the Franconian alabaster deposits of the Steigerwald region E and SE of Würzburg, different from all other European deposits so far investigated, are the only with isotopic fingerprints compatible with both those of the Rimini and Riemenschneider corpuses, the most discriminating parameters being strontium and sulphur isotopes.

The only notable exception is the head of Saint Peter as part of the Rimini crucifixion (Fig 3P) with a clearly Permian sulphate isotope composition that can be linked to the Harz alabaster quarries near Nordhausen. The Saint Peter was headless when Swarzenski acquired it in 1913 [3, 4] for the Liebieghaus and was combined with an existing head, most likely dating from the 19^th^ century.

The Ickelheim alabaster is the only of the investigated Franconian deposits to show the distinctive macroscopic features observed for the Rimini sculptures (Fig 3). Also, the dimensions of the artwork are compatible with the use of gypsum nodules as stated by our 18^th^ century source [39] from the Ickelheim quarries. The duration of four centuries of exploitation of the Ickelheim quarries mentioned by Hofmann in 1757, though to be taken with care, would include the production phases of the Rimini and Riemenschneider workshops. We thus conclude that the Ickelheim quarries are the most likely source of supply for both.

Concerning the supply and location of the Rimini workshop, two new scenarios come into question:

1. **The workshop was situated in the Southern Netherlands or in Northern France and was exclusively supplied with Franconian alabaster**. This scenario is based on the current consensus on the influence of Flemish painting on the style of the Rimini sculptures, notably of the Master of Flemalle [4], and on the scarce hints on the Parisian art market for the trade of the Rimini productions, notable for the Wrocław group [11].
2. **The Rimini workshop was situated near the Franconian deposits and used exclusively the material easily available near its doorstep**, as it is evident for the Riemenschneider alabaster production. The Master of Rimini might, in this case, have received his education in the Franco-Flemish sphere or been strongly inspired by the art of this region, marking his iconography and style along with other sources of influences, particularly from Central European art [14].

We cannot, based on isotope fingerprinting alone, decide between both scenarios, yet our findings prove a voluntary choice of the material, either for aesthetic or for practical/economic reasons.

The first scenario would imply a transport of more than 600 km from the Franconian quarries to the Netherlands, to Bruges, suggested by Kim Woods as a possible location of the workshop [1, 2]. Trade of raw materials over long distances is now well documented [46], for English alabaster [19, 47], the French Alpine deposits [19] and, to a lesser extent, of Spanish material (notably for the Beuda quarries [19]). The Main and Rhine rivers may have served for riverine transport, the Ickelheim quarries being situated 37 km from the Main river (Fig 1), the latter flowing to the Rhine, navigable as far as the Southern Netherlands since antique times [48]. Furthermore, the dimensions of the blocs are rather modest, all below 60 cm for our corpus, facilitating transport, a specificity of the Rimini sculptures pointed out by Legner [4].

However, the first scenario raises a number of questions: If the workshop was situated in the Southern Netherlands or in Northern France, why did it not use other, more readily available material, e.g. English alabaster from the East Midlands or French Alabaster from the Alpine deposits, known to have transited at this time to and through France [19]? Why did the Master of Rimini choose Franconian alabaster for his workshop? Here, we may suggest that he knew these deposits, perhaps due to German roots, and for some (aesthetic, relational, economic?) reasons clung to them during the whole lifetime of his workshop. We have other examples of such a conservatism in the choice of a specific material, e.g., fifty years later, the sculptor Martin Claustre, originating from Grenoble, who continued using the alpine alabaster of Notre-Dame-de-Mésage, long after having left the Dauphiné region [49]. The last question is the most intriguing one: If a Europe-wide export of Franconian alabaster existed, why do we find its fingerprints in none of the other medieval and early modern European artworks published so far [19, 20]? One can argue that the total number of analyses is still quite limited, compared to the existing alabaster European artwork of the late Middle Ages, and that there are some inevitable biases of sampling. Notably, the 15^th^ century alabaster sculpture from the Low Countries was in large parts destroyed by the iconoclast Reformation movements of the 16^th^ century and is therefore inaccessible to investigation. German alabaster sculpture is so far underrepresented in our corpus. Nonetheless, the 15^th^ cent. use of Franconian alabaster appears, at the present state of our research, focused on and exclusive for two workshops, plus eventual followers, one of them, the Riemenschneider workshop, being situated with certitude in the Franconian region.

This provides some arguments for the second scenario, a regional anchoring of the Rimini workshop in Southern Germany. In this case, why does the style and iconography of the Rimini group show such close links with Flemish art, alongside other influences [4, 9, 14]? Before the second half of the 15^th^ century, artist biographies are rarely known with sufficient detail to reconstruct their mobility. Hypotheses of artist itinerancy were so far largely based on stylistic considerations and the idea of non-resident artists wandering from town to town in Europe, thus transmitting new stylistic tendencies (“Wanderkünstler”). This concept was later qualified as a “scientific myth” for individual sculptures [50], even if it still holds for cathedral building lodges. Later and better documented biographies show, that, indeed, Flemish artists in the late 15^th^ and the early 16^th^ century migrated all over Europe, including Spain [51], England [52] and Germany [53], thus disseminating the Flemish style and making it the international reference of the time. A prominent example of a migrating albeit not itinerant artist is the sculptor Nicolas Gerhaert born in Leyden around 1420 who installed a workshop in Strasbourg before 1463, and accepted in 1467 an offer of the Emperor and moving to the imperial residence of Wiener Neustadt where he died in 1473 [54].

The Master of the Rimini Altarpiece could be considered as an earlier example of such international mobility. Yet, the constancy of supply clearly contradicts the hypothesis of an itinerant artist [50] introduced at the beginning of the Rimini research by Swarzenski [3] and Körte [55] and confirms the prevailing theory of a geographically stable, highly specialized export workshop. We may postulate that it was run by a sculptor who, having received his education or artistic inspiration in the Flemish sphere, installed his workshop in Southern Germany. Like later Nicolas Gerhaert in Strasbourg [56] and the numerous Flemish artists working in Spain [57, 58] in the second half of the 15^th^ century, he might have sought to escape the constraints of the rigid guild system of towns in the Low Countries like Bruges which throttled artistic innovation [57]. Furthermore, as Jolly states for Flemish sculptor’s migration to early Renaissance Germany, another motivation might have been the readily available raw material that was non-existent in the Netherlands [53].

A potential candidate for the Rimini workshop’s location is the town of Nuremberg, 52 km east from the identified alabaster deposits (Fig 1), where we have evidence of a long-lasting tradition of alabaster carving. In Nuremberg, up to the end of the 17^th^ century this profession was considered as “free art”, facilitating the access for any gifted artisan without requirement of a masterpiece [59], as opposed to “sworn crafts”, the latter being overseen by a master [60]. The craftsmen’s revolt of 1349 had led to an abolition of the guild system, Nuremberg’s Lesser Council taking the entire governance of the local craft and trade [61]. Only in 1698 a guild of alabastermen (“Alabasterer”) was founded leading to a culmination of activity till the 1720s [59]. By the end of the 18^th^ century, this profession seems to have virtually vanished [59]. The oldest explicit mention of an alabaster carver in Nuremberg dates from 1441 [62] when a Martin Guldein became citizen, proving that, in the early 15^th^ century, specialized artists were active in the region. They probably already relied on local supply, thus corroborating Hofmann’s statement of a long-lasting activity for the Ickelheim quarries [39]. He mentions that “in the past, frequently 40 to 50 cartloads of such stones” from the Ickelheim vineyards “parted to the Imperial town of Nuremberg, a meeting place of artists”. The attractiveness of the Imperial City of Nuremberg as a leading centre of craft production is indeed directly related to its Lesser Council’s policy [61]. From the second half of the 14^th^ century onwards, it facilitated, through a strong reduction of the regulatory burden, in particular for the “free arts” including sculpture, the immigration, settlement and work of a large number of talented artists [61].

A vast network of commercial relations radiated from the postulated highly productive South German production centre of alabaster sculpture both to the South, across the Alps to Northern Italy, and, eventually via Paris, to Northern France and to other European capitals. This is illustrated by the altarpieces of Rimini and Isola Bella (formerly in Milano), Saint-Omer and, potentially, the lost ensemble of Arras, the altarpieces of Schwabstedt and Schwerte in Northern Germany, as well as the crucifixion group from Wrocław (Fig 1). The Imperial City of Nuremberg might have favoured the development of such a network. At the crossroads between Central and Western Europe, between the Hanse and Northern Italy, the town benefited from imperial protection and freedom of trade since the 12^th^ century [63]. It developed a farsighted policy of free trade agreements and merchant mobility with all major trade centres of Europe, from Lübeck to Venice, Bruges to Cracow and Wrocław [63].There have been strong economic and artistic links between Nuremberg and Wrocław, facilitating direct art trade of Franconian artwork delivered to Silesia [61] even if the Wrocław alabaster group seems to have transited through the anonymous art market via Paris. Such modern forms of the art market developed, including art centres in Southern Germany, in parallel to traditional lines where art trading agents linked workshops to wealthy patrons [64]. As stated by Woods [1] the outstanding quality of the Master of Rimini’s personal production met the demands of the upper end of the market, similarly to the more or less contemporaneous paintings of Van Eyck. Direct commissions from high-ranking clients like the Milanese Borromei and, most likely, the Malatestas of Rimini [1] could have prevailed for the few conserved masterpieces whereas the workshop production, including serial production of devotional objects, eventually also by followers, would rather be intended for the open market [4]. This could also explain why none of the known masterpieces of the workshop were initially situated in the Nuremberg and even Franconian region whereas several lesser artworks, mainly Pietàs as our examples from Würzburg and Bad Mergentheim (Fig 3F and 3G), can be traced back to churches in Southern Germany [40, 65].

Our results provide a means to better constrain the large and somewhat protean Rimini alabaster corpus as it is obviously possible to identify unambiguously the preferred material used by this workshop. They should also help to geographically focus future historical research targeting so far unreported written evidence of the workshop’s activities. Our study illustrates how independent archaeometric methods can shed a new light on the art historical discussion on artist mobility and art trade routes prior to the mid-15^th^ century in a context of still sparse written sources on artist identities and *vitae* and the more than patchy records of individual art trade transactions. Stylistic analyses, inevitably subjective to some extent, have been the main arguments for geographically situating the Rimini workshop with its pan-Europe radiance, with quite contradictory results. Even if the material sciences cannot provide an ultimate answer to the enigma of the Master of the Rimini Altarpiece, we provide corroborating evidence on a territorial anchorage within or a strong link to Southern Germany, thus re-opening a debate that otherwise seemed to have come to a dead-end.

## Supporting information

S1 File (PDF)
