# Supplementary material for: A pan-European art trade in the late middle ages: Isotopic evidence on the master of Rimini enigma
Source: PLoS One. 2022 Apr 12;17(4):e0265242. doi: 10.1371/journal.pone.0265242 (PMC9004741; doi:10.1371/journal.pone.0265242)
Supplement: S1 File — (PDF) [file pone.0265242.s001.pdf]

Supporting Information

Title
A pan-European art trade in the Late Middle Ages: Isotopic evidence on the Master of Rimini enigma

Authors
W. Kloppmann, L. Leroux, Ph. Bromblet, P.-Y. Le Pogam, A.T. Montech, C. Guerrot

S1 Appendix
Complementary findings on the 1431 purchase of the Wrocław alabaster group by the abbot Jodocus for the Augustinian monastery of our Lady of the Sand from a French merchant

Only two written sources from the period of activity of the Rimini workshop are known referring to international trade of alabaster sculptures potentially linked to the Rimini group. One is part of the records of Abbot Jodocus of the Augustinian monastery of Wrocław who acquired in 1431 a Crucifixion group from a "Parisian" merchant. This source is all the more noteworthy as one surviving sculpture from this Crucifixion has been identified, the Swooning of the Virgin group, now conserved in the National Museum of Warsaw. It is included in the corpus of our study (Fig. 2C).

The obscure expression "*parysiis in montanis*" referring to the origin of the sculpture has been subject to discussion since this source was first related to the Wrocław Swooning Virgin by Scheyer in 1933[1]. He explains the phrase "*qui mercator affirmavit praefatam imaginem crucifixi sculptam parysiis in montanis*" in the following way: The Parisian merchant claims a Parisian origin of the sculpture, "*parysiis in montanis*" referring to the topography of the "hilly" Paris and perhaps to the Montmartre gypsum deposits, even if he judges this hypothesis as unlikely. All later literature cites the expression after Scheyer (1933).

Based on an earlier reference and on the original manuscripts conserved in the University Library of Wrocław (references IV Q 205, ff. 37v-64 chart. and V F 200b, pp. 69-111 chart.), we propose a new reading of this passage, crucial for the Rimini research, and postulate that the enigmatic expression is in fact a transcription error.

Indeed, after Stenzel (1839) [2], p. 223, the original manuscript of Jodocus has been lost and two transcriptions exist, one from 1470 by the successor of Jodocus and the other from the beginning of the 17th century. Stenzel edits the older version and here the decisive phrase reads: "*qui mercator affirmavit, prefatam ymaginem crucifixi sculptam* <u>*in Parisius cum montanis*</u>" (S1 Fig.). This expression "*cum montanis*" could refer to the physical support of the Crucifixion group, in form of mountains, so that the phrase can be translated as "This merchant confirmed that the crucified Christ's representation had been sculpted, together with mountains/with a mountain-shaped base, in Paris". This would mean that "*montanis*" refers to a Calvary-type arrangement for the Wrocław ensemble, similar to the alabaster Crucifixion conserved in the Halberstadt cathedral (Germany), made by a sculptor inspired by the Rimini Crucifixion or another altarpiece from the Rimini workshop [3] (S3 Fig.). In Halberstadt, the sculptures are based on a wooden structure in form of hills and assembled as a "Kalvarienberg", a Calvary. It is likely that this altarpiece is still in its original position and in its original order[3]. This reading of "*montanis*" as "mountain-shaped support" for the Wrocław group is supported by the following passages in Stenzel's version, mentioning, for another ensemble of sculptures: "*montana minerarum, artificialiter et subtiliter multum facta*", "mineral mountains, made with great art and refinement", supporting groups of small figures, which have later collapsed by negligence of the sacristans.

In the following, we present the original Latin text of the 1470 transcription of Jodocus' lost manuscript in Stenzel's edition as well as an English translation, based on the translation

into German kindly provided by Prof. Dr. Gerhard Weilandt (University of Greifswald) and on the initial French translation kindly rendered by Françoise Lami, Orléans.

Item, anno domini MCCCCXXXI, circa festum sancti Johannis baptiste, dominus Jodocus abbas emit a quodam mercatore de Parisius tabulam cum crucifixo de alabastro laboratum cum suis attinenciis, sitam in altari s. Augustini pro XL. florenis Ungaricis, qui mercator affirmavit, prefatam ymaginem crucifixi sculptam in Parisius cum montanis sed tabulam idem mercator Wratislavie ad eandem ymaginem fieri disposuit, pro qua X. marcas denariorum exposuit. Et eodem anno, in die s. Elyzabeth, idem dominus Jodocus abbas solvit Johanni Crommendoff aurifabro XX. marcas latorum grossorum in auro racione ejusdem tabule et laborum circa eandem, ut patet in libro annotacionum ejusdem. Item, eodem anno idem dominus Jodocus abbas apud quendam Smedchen, civem Wralislaviensem, emit quedam montana minerarum, artificialiter et subtiliter multum facta, cum tribus regibus et montanorum fossoribus et Christofero et aliis ymaginibus parvis et subtilibus, pro XXVI. florenis Ungaricis, que montana postea per incuriam custodum ecclesie sunt collapsa, confracta et deperdita.

"Likewise, in the year of the Lord 1431, around the feast of Saint John Baptist, the Abbot Jodocus bought from a certain merchant from Paris an altar table/shrine with the crucified Christ, which was made of alabaster, with associated parts, situated at/in the altar of Saint Augustine, for 40 Hungarian guilders/florins. This merchant confirmed that the crucified Christ's representation had been sculpted, together with mountains/with a mountain-shaped base (cum montanis), in Paris, but this merchant had the altar table/shrine for this sculpture made in Wrocław, for which he spent 10 marks of pennies. And in the same year, on the day of Saint Elisabeth, the same Abbot Jodocus paid Johann Crommendorff, goldsmith, 20 marks of thick groats/Groschen in gold on the account of this altar table/shrine and the related works, as it is written in the book of his notes. Likewise, in the same year, the Abbot Jodocus bought from a certain Smedchen, citizen of Breslau, certain mineral mountains (montana minerarum), made with great art and refinement, with the Three Kings and miners and Saint Christopher and other small and delicate representations for 26 Hungarian guilders/florins. These mountains/mountain-shaped base (montana) later collapsed, broke and were lost due to the carelessness of the sacristans of the church."

In the 1609 copy, the passage "*Parisius cum montanis*" initially read, like in the original, "*parisiůs ců (m) mó (n)tanis*" but was changed by a later hand into "parisiis in montanis" by transforming the "ů" of "parisiůs" into a double "i" and the "c" of "ců (m)" into an "i" and the "ů" into an "n" (S2 Fig.).

Scheyer refers to an 18th century, copy of the text, „*Chronicum compendiosum complectens Canoniam Wratislavienscm in Arena . . . ab anno 1108 usque ad annum 1726 Balthasare Antonio Biener* " (Breslau Diöz. Archiv V, 4). This version takes over the corrections to the 1609 manuscript. Furthermore, it seemingly introduces new transcription errors: Scheyer's version also mentions an altar dedicated to "Augustus" ("*Augusti*") which is an obviously erroneous copy of "*altari s. Augustini*" dedicated to Saint Augustin, correctly spelled in the 1609 copy.

These findings imply that the passage clearly refers to a Parisian origin of the Wrocław group, the suspicious expression "*in montanis*" being identified as transcription error. Even though, the affirmation of the merchant is to be taken with precaution, as it might have been in his interest to mention Paris as prestigious centre of art production.

It further suggests that the Wrocław group was part of a Calvary and that the supporting wooden structure was delivered together with the alabaster figures whereas the corresponding shrine (tabula) was later produced in Wrocław. This sheds new light on the exportation and the arrangement of large alabaster ensembles as the one in Halberstadt but also the Rimini Crucifixion now conserved in Frankfurt, for which a Calvary-type arrangement could be equally envisaged.

References

1. Scheyer E. Eine Pariser Alabaster-Gruppe um 1430. Schlesiens Vorzeit in Schrift und Bild Jb d Schlesischen Museums f Kunstgewerbe und Kunstaltertümer. 101933. p. 35-42.

2. Stenzel GA. Scriptores rerum Silesiacarum; oder, Sammlung schlesischer Geschichtschreiber, namens der Schlesischen Gesellschaft für vaterländische Cultur [afterw.] (des Vereins für Geschichte und Alterthum Schlesiens) herausg. von G.A.Stenzel. Breslau: Josef Max & Komp.; 1839. 505 p.

3. Jopek N. Studien zur deutschen Alabasterplastik des 15. Jahrhunderts. Werner F, editor. Worms: Werner; 1988. 219 p.

4. Guillot de Suduiraut S. Maître du retable de Rimini (entourage du). In: Bresc-Bautier G, editor. Les sculptures européennes du musée du Louvre. Paris: Hazan; 2006. p. 352-3.

5. Kautzsch R. Die Alabastergruppe der trauenden Frauen im Schlesischen Museum für Kunstgewerbe. Jahrbuch des Schlesischen Museums für Kunstgewerbe und Altertümer1919. p. 176-84.

6. Kriegseisen J, Lipinska A, editors. Cat Gdansk Materia światła i ciała -Matter of Light and Flesh. Alabaster in the Netherlandish Sculpture of the 16th and 17th centuries, Exhibition Catalogue, National Museum in Gdańsk, 15.11.2011 – 15.03.2012, Gdańsk 2011. Gdansk Muzeum Narodowe w Gdańsku; 2011.

7. Swarzenski G. Deutsche Alabasterplastik des 15. Jahrhunderts. Städel Jahrbuch. 1921;1:167-213.

8. Heisig I. Pieta. In: von der Bank M, Heisig I, editors. Mittelrhein-Museum Koblenz, Auswahlkatalog. Petersberg: Michel Imhof Verlag; 2017. p. 28-9.

9. Weschenfelder K. Mittelrhein-Museum Koblenz. Wallraf-Richartz-Jahrbuch. 1991;52:358-.

10. Gröber K. Die Kunstdenkmäler von Unterfranken und Aschaffenburg. München1914. 110-2 p.

11. Kunz T. Bildwerke nördlich der Alpen und im Alpenraum 1380 bis 1440. Kritischer Bestandskatalog der Berliner Skulpturensammlung. Petersberg: Michael Imhof; 2019.

12. Woods K. The Master of Rimini and the tradition of alabaster carving in the early 15th century Netherlands. Nederlands Kunsthistorisch Jaarboek. 2012;62(1):56-83.

13. Woods KW. Cut in Alabaster: a Material of Sculpture and its European Traditions 1330-1530. Turnhout: Brepols; 2018. 422 p.

14. Legner A. Der Alabasteraltar aus Rimini. Städel-Jahrbuch. 1969;Neue Folge, Band 2:101 - 68.

15. Hood A. A Rare Medieval Alabaster Los Angeles: J. Paul Getty Museum; 2015 [cited 2019 March 04, 2019]. Available from: http://blogs.getty.edu/iris/this-just-in-a-rare-medieval-alabaster/.

16. Guillot de Suduiraut S. Quatres apôtres 47 a, b, c, d. In: Le Pogam PY, editor. Les premiers retables. Paris: Officina Libraria; 2009. p. 175-7.

17. Gil M, Nys L. Saint-Omer gothique. Les arts figuratifs à Saint-Omer à la fin du Moyen Âge 1250-1550 : peinture - vitrail - sculpture - arts du livre,. Valenciennes: Presses Universitaires de Valenciennes; 2004. 534 p.

18. Bier J. Riemenschneider's St. Jerome and His other Works in Alabaster. The Art Bulletin. 1951;33(4):226-34. doi: 10.2307/3047371.

19. Husband TB. Tilman Riemenschneider and the tradition of alabaster carving. Studies in the History of Art. 2004;65:64-81. PubMed PMID: CCC:000236599900007.

20. Großmann O. Eine Madonna von Riemenschneider? In: Rauch C, editor. Hessen-Kunst Kalender für Kunst- und Denkmalpflege 4 Jahrgang. Marburg a. L.: Verlag von Adolf Ebel; 1909. p. 31-2.

21. Michel A. Les récentes acquisitions du département de la sculpture (Moyen Âge, Renaissance et Temps modernes) au musée du Louvre. Gazette des Beaux-Arts. 1906;3e ser., 35, vol. 1:393-414.

22. Chapuis J, Baxandall M, Borchert T-H, Husband TB, Kemperdick S, Krohm H, et al. Tilman Riemenschneider: Master Sculptor of the Late Middle Ages. New Haven and London: National Gallery of Art (Washington), The Metropolitan Museum of Art (New York), Yale University Press; 1999.

23. Bier J. Tilmann Riemenschneider: His Life and Work. Lexington, Kentucky: University Press of Kentucky; 1982. xiii +128 p.

24. Lichte C, Kneise U. Die Riemenschneider Sammlung im Mainfränkischen Museum Würzburg. München: Prestel; 2014. 96 p.

25. Borchert T-H. Tilman Riemenschneider. The Annunciation : A new addition to the corpus of works in alabaster. London: Daniel Katz Ltd.; 2012.

26. Hofmann D. Beschreibung eines Alabaster-Steinbruchs in Francken. In: Delius HF, editor. Fränkische Sammlungen von Anmerkungen aus der Naturlehre, Arzneygelahrheit, Oekonomie und den damit verwandten Wissenschaften. Nürnberg: Monath, G. P.; 1757. p. 129-49.

27. Kloppmann W, Leroux L, Bromblet P, Guerrot C, Proust E, Cooper AH, et al. Tracing Medieval and Renaissance Alabaster Works of Art Back to Quarries: A Multi-Isotope (Sr, S, O) Approach. Archaeometry. 2014;56(2):203-19. doi: 10.1111/arcm.12008.

28. Kloppmann W, Leroux L, Bromblet P, Le Pogam PY, Cooper AH, Worley N, et al. Competing English, Spanish, and French alabaster trade in Europe over five centuries as evidenced by isotope fingerprinting. Proceedings of the National Academy of Sciences. 2017;114(45):11856–60.

29. Bromblet P, Kloppmann W, Leroux L, Le Pogam PY, Muñoz del Pozo A, Morte García C. L'albâtre de Beuda (Gérone, Catalogne, Espagne), un matériau marqueur de la sculpture gothique en France méridionale révélé par les analyses multi-isotopiques (S, O, Sr). ArcheoSciences, revue d'archéométrie. 2021;44(2):175-88.

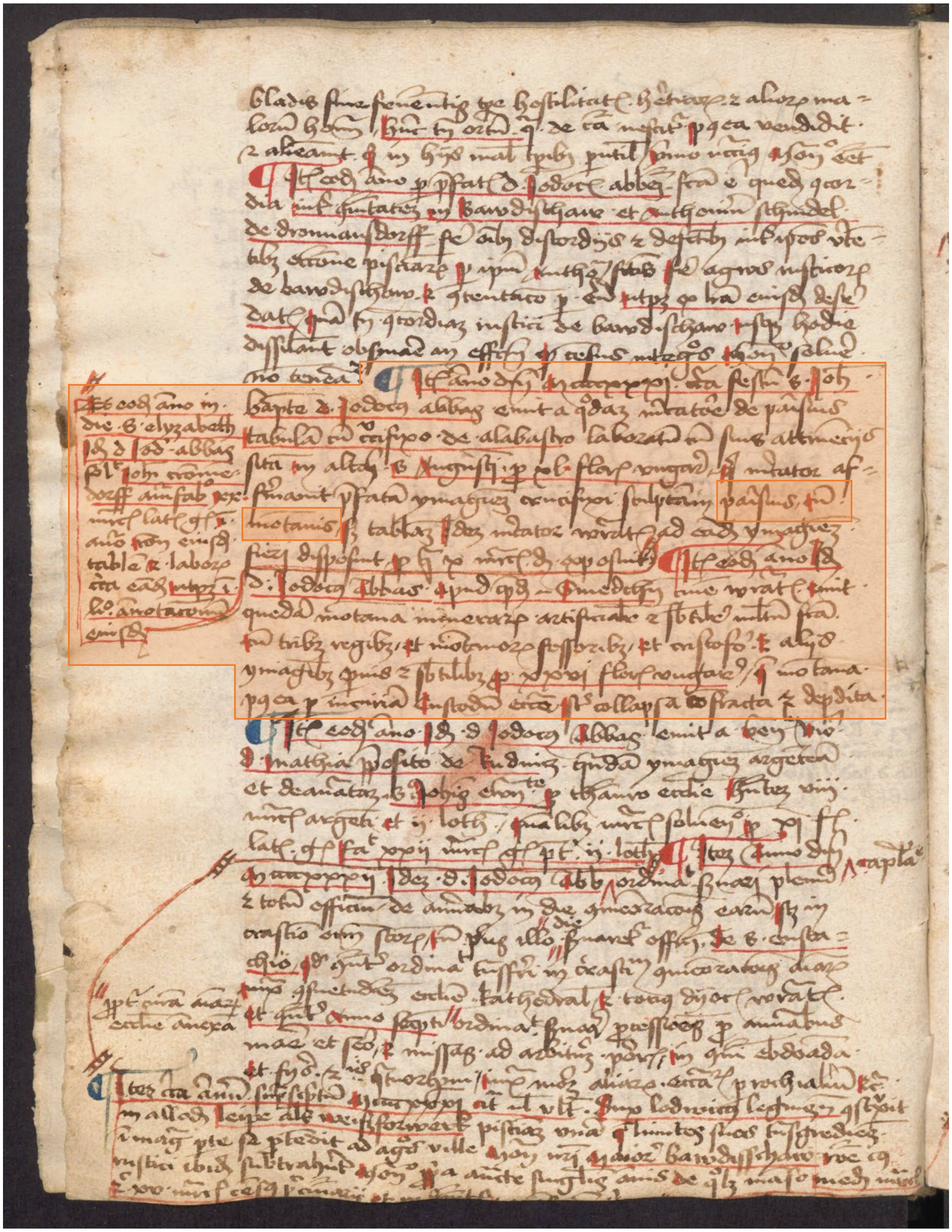

S1 Fig. Manuscript IV Q 205, ff. 37v-64 chart., the *Chronica abbatum Beatae Mariae virginis in Arena* from 1470, University Library of Wrocław, by the hand of Abbot Benedict Johnsdorff, successor of Jodocus Czeginhals. The passage concerning the acquisition of the alabaster Calvary in 1431 and the subsequent purchase of a group of sculptures arranged on a support in the form of mountains is highlighted. The decisive passage (double highlighted) on the origin of the alabaster group reads here “parisius cum montanis”.

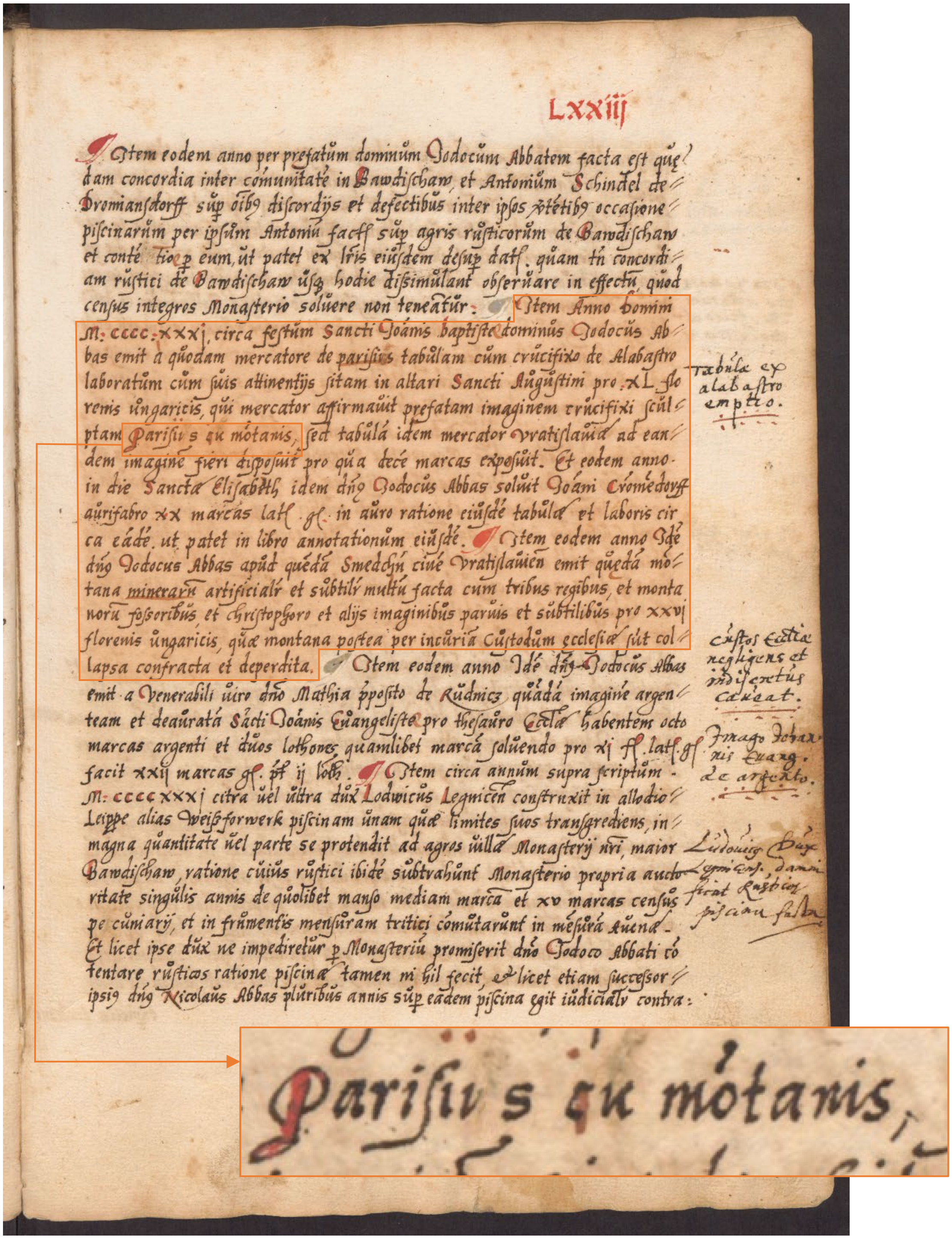
LXXIIII

Item eodem anno per prefatum dominum Jodocum Abbatem facta est quę-
dam concordia inter comunitate in Bawdischaw et Antonium Schindel de
Dromiansdorff sup oib9 discordijs et defectibus inter ipsos ortetib9 occasione
piscinarum per ipsum Antoniu facti sup agris rusticorum de Bawdischaw
et conte tio p eum, ut patet ex lris eiusdem desup datis. quam tn concordi-
am rustici de Bawdischaw usq hodie dissimulant observare in effectu quod
census integros Monasterio soluere non teneatur. Item Anno domini
M. cccc. xxxi. circa festum Sancti Joannis baptiste dominus Jodocus Ab-
bas emit a quodam mercatore de parisiis tabulam cum crucifixo de Alabastro
laboratum cum suis attinentijs sitam in altari Sancti Augustini pro XL flo-
renis ungaricis, qui mercator affirmavit prefatam imaginem crucifixi scul-
ptam Parisiis s eu motanis, sed tabula idem mercator Vratislauia ad ean-
dem imagine fieri disposuit pro qua dece marcas exposuit. Et eodem anno
in die Sancta Elisabeth idem dns Jodocus Abbas soluit Joani Cromedorff
aurifabro XX marcas lat. gr. in auro ratione eiusde tabule et laboris cir-
ca eade. ut patet in libro annotationum eiusde. Item eodem anno Ide
dns Jodocus Abbas apud queda Smedchin cive Vratislauien emit queda mo-
tana minerarum artificialr et subtilr multu facta cum tribus regibus, et monta
noru fossoribus, et christophoro et alijs imaginibus parvis et subtilibus pro XXVI
florenis ungaricis, quae montana postea per incuria Custodum ecclesiae sut col-
lapsa confracta et deperdita. Item eodem anno Ide dns Jodocus Abbas
emit a Venerabili viro dno Mathia pposito de Rudnicz quada imagine argen-
team et deaurata Sancti Joannis Evangeliste pro thesauro Eccle habentem octo
marcas argenti et duos lothones quamlibet marca soluendo pro XI fl. lat. gr.
facit XXII marcas gr. pt ij loth. Item circa annum supra scriptum
M. ccccXXXI citra vel ultra dux Lodwicus Legnicen construxit in allodio
Leippe alias Weissforwerk piscinam unam quae limites suos transgrediens, in
magna quantitate vel parte se protendit ad agros villa Monasterij nri, maior
Bawdischaw, ratione cuius rustici ibide subtrahunt Monasterio propria aucto-
ritate singulis annis de quolibet manso mediam marca et XV marcas census
pecuniarij, et in frumentis mensuram tritici comutarunt in mesura Auene.
Et licet ipse dux ne impediretur p Monasteriu promiserit dno Jodoco Abbati co-
tentare rusticos ratione piscinae tamen nihil fecit, et licet etiam successor
ipsi9 dns Nicolaus Abbas pluribus annis sup eadem piscina egit iudicialr contra:

Tabula ex alabastro emptio.

Custos Eccl[ia]e negligens et indiscretus caveat.

Imago Joannis Evang. de argento.

Ludovicus Dux Legnicens. damnificat Rusticos piscina facta

Parisiis s eu motanis

S2 Fig. Manuscript IV F 200b, pp. 69-111 chart., University Library of Wrocław, a copy from 1609 of the *Chronica abbatum Beatae Mariae virginis in Arena.* The same passage as in S1 Fig. concerning the alabaster purchase is highlighted. The passage on the provenance is double highlighted.

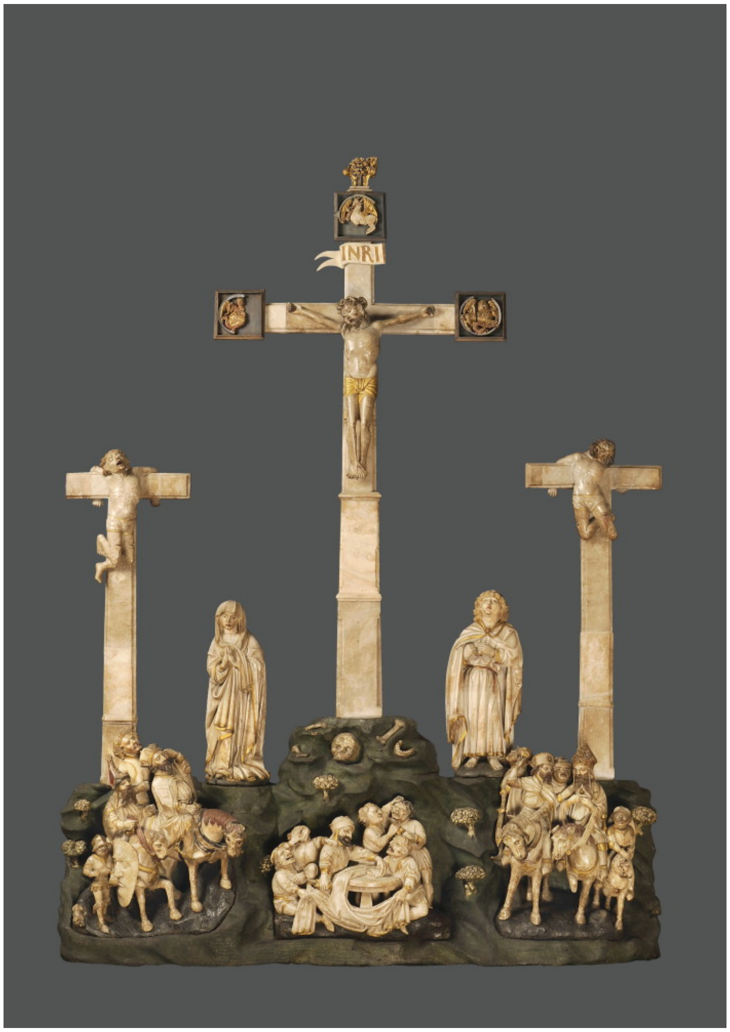

S3 Fig. Alabaster Crucifixion mounted in a Calvary arrangement, Halberstadt Cathedral, around 1460, published with permission of the Landesamt für Denkmalpflege und Archäologie Sachsen-Anhalt under CC BY 4.0 license (original © Landesamt für Denkmalpflege und Archäologie Sachsen-Anhalt, Juraj Lipták, 2022)

S1 Table. Analyzed artwork. Artwork attributed to the Rimini group and to Tilman Riemenschneider's workshop analysed in this study, supposed period of creation, dimensions, original situation of the artwork if known, current situation/collection and context.

Artwork	Period	Dimen-sions (cm)	Original monument/ situation	Current situation	Context/ Literature	Figure
Swoon of the Virgin group	~1430	H 46.5	Santa Maria delle Grazie church (Rimini, Italy)	Liebieghaus Skulpturensammlung, (Frankfurt, Germany) Inv. 402	Master of Rimini. Crucifixion altarpiece initially (?) in the church Santa Maria delle Grazie (Rimini, Italy) probably since its consecration in 1430, recorded since 1580 (2). Acquired for the Liebieghaus by Swarzenski in 1913. (2, 3, 6, 7)	Fig.2A
Swoon of the Virgin	~1430	H 29, W 18, D 10	Unknown	Musée du Louvre (Paris, France), Inv. RF 1639	Master of Rimini group. Part of a crucifixion. Donation to the Louvre in 1916 [4]	Fig.2B
"Three Maries" (Swoon of the Virgin)	~1430	H 46, W 23	Church of our Lady of the Sand (Wrocław, Poland)	National Museum, (Warsaw, Poland), Inv. Śr.402	Master of Rimini group. Part of a Crucifixion acquired in 1431 for the church of our Lady of the Sand in Wrocław (Poland) by the Abbot Jodocus of the Wrocław Augustinian friary from a Parisian merchant. After the secularization of the Silesian monasteries in 1810 the group came first to the former Schlesisches Museum für Kunstgewerbe und Altertümer, Wrocław, after WWII to the National Museum in Warsaw [1, 3, 5-7],	Fig.2C
Pietà	~1450	H 48, W 41, D 14	Unknown	Rijksmuseum (Amsterdam, Netherlands), Inv. BK-NM-11667	Master of Rimini group. Donation of A. Pit, Amsterdam, 1904	Fig.2D
Pietà	First half 15th cent.	H 26,5	Unknown	Mittelrheinmuseum (Koblenz, Germany), Inv. P 1990/13	Master of Rimini group. Acquired in 1990 from the art market (via Christie's, London, auction April 18, 1989, from A. Neuhaus, Würzburg, Germany)[8, 9]. Virtually identical to the Pietà group in Würzburg (Museum am Dom, Cathedral), originating from Großwenkheim, north of Würzburg.	Fig.2E
Pietà	Around 1430	H 25 cm	Pfarrkirche Mariae Himmelfahrt (Großwenkheim, part of Münnerstadt, district Bad Kissingen, Germany) before 1914	Museum am Dom (Würzburg, Germany), Inv. 491	Master of Rimini group. Documented in 1914 [10] in the church of Großwenkheim, Münnerstadt (Germany) in a strongly painted version, later restored. Virtually identical to the Koblenz Pietà (Mittelrheinmuseum).	Fig.2F
Pietà	Around 1430	H 36, D 10.5	Marienkirche (former Dominican, then Teutonic church), Bad Mergentheim, Germany, before 1853 (?)	Deutschordensmuseum (Bad Mergentheim, Germany)	Loose stylistic connection to the Master of Rimini group but contemporaneous [11]. Documented in the 19th cent. in the Dominican church (constructed in the late 14th cent.) of Bad Mergentheim, later owned by the Teutonic Order.	Fig.2G
Apostle Saint Paul	~1430	H 44.5	Santa Maria delle Grazie church (Rimini, Italy)	Liebieghaus Skulpturensammlung, (Frankfurt, Germany) Inv. 406	Master of Rimini. Crucifixion altarpiece initially (?) in the church Santa Maria delle Grazie (Rimini, Italy) probably since its consecration in 1430, recorded since 1580[12]. Acquired for the Liebieghaus by Swarzenski in 1913 [7, 12-14]	Fig.2H
Apostle	~1430	H 46.1	Santa Maria delle Grazie church (Rimini, Italy)	Liebieghaus Skulpturensammlung, (Frankfurt, Germany) Inv. 409	Master of Rimini. Crucifixion altarpiece initially (?) in the church Santa Maria delle Grazie (Rimini, Italy) probably since its consecration in 1430, recorded since 1580[12]. Acquired for the Liebieghaus by Swarzenski in 1913 [7, 12-14]	Fig.2I
Apostle Saint Andrew	~1430	H 46.3	Santa Maria delle Grazie church (Rimini, Italy)	Liebieghaus Skulpturensammlung,	Master of Rimini. Crucifixion altarpiece initially (?) in the church Santa Maria delle Grazie (Rimini, Italy) probably since its	Fig.2J

				(Frankfurt, Germany) Inv. 417	consecration in 1430, recorded since 1580[12]. Acquired for the Liebieghaus by Swarzenski in 1913 [7, 12-14]	
Apostle Saint Peter	~1430	H 9.5 (head)	Unknown	Liebieghaus Skulpturensammlung, (Frankfurt, Germany) Inv. 418	Replacement of the head of the Saint Peter statue of the Rimini altarpiece, already headless when Swarzenski acquired the Rimini Altarpiece for the Liebieghaus. Alabaster, probably 19th cent.	Fig.2Q
Apostle Saint Philip	~1420-1430	H 43	Unknown	J.P. Getty Museum (Los Angeles, USA), Inv. 2015.58	Master of Rimini group. Formerly collections of Ottmar Strauss, Cologne, and Oskar and Ilse Mulert, Frankfurt (Germany) acquired by the J.P. Getty Museum in 2015. [15]	Fig.2K
Apostle	~1430	H 22.9; W 8.6; D 5	Unknown; supposedly Saint-Omer Cathedral (Saint-Omer France)	Musée de l'Hôtel Sandelin (Saint-Omer, France), Inv. 2911.3	Master of Rimini group. Currently thought to have been part of an Altarpiece in the Saint-Omer Notre-Dame Cathedral, ordered in 1429 by the Canon Gauthier Ponche, similar geographic context as the lost altarpiece of the St. Vaast Abbey, Arras (1431). Donated to the museum in 1840, earlier history unknown [16, 17].	Fig.2L
Apostle	~1430	H 24.2; W 9.6; D 5.2	Unknown; supposedly Saint-Omer Cathedral (Saint-Omer France)	Musée de l'Hôtel Sandelin (Saint-Omer, France) Inv. 2911.4	Master of Rimini group. Currently thought to have been part of an Altarpiece in the Saint-Omer Notre-Dame Cathedral, ordered in 1429 by the Canon Gauthier Ponche, similar geographic context as the lost altarpiece of the St. Vaast Abbey, Arras (1431). Donated to the museum in 1840, earlier history unknown [16, 17].	Fig.2M
Apostle	~1430-40	H 39; W 16; D 10	Unknown	Musée du Louvre (Paris, France) Inv. RF 4402	Master of Rimini group (entourage). Statue of an apostolic college, integrated in an altarpiece. Acquired in 1922 on the art market. [4]	Fig.2O
Apostle	~1430	H 29, W 12 (base: H 5.5, W 9, D 8.8)	Saint Victor Church (Schwerte, Germany)	Saint Victor Church (Schwerte, Germany)	Interesting case of reuse of a series of 15th century alabaster apostles (eight preserved) around a central enthroned Christ. They were integrated in a typical wooden carved Antwerp altarpiece, installed in 1523 in the Saint Viktor church in Schwerte (North Rhine-Westphalia, Germany), commissioned in 1521 by the Franciscan community of Dortmund (Germany). The style of the alabaster apostles is typical for the Rimini Workshop. The atypical polychromy of the apostles corresponds in style and motives to the wooden Antwerp altarpiece and dates most likely from the integration of the apostles in the wooden retable. The one century older alabaster ensemble might provide from a lost altarpiece in Schwerte or was exported together with the wooden retable (pers. comm. N. Gliesmann)	Fig.2P
Annunciation group: Virgin	~1495-1500	H 54, W 36.5, D 17	Supposedly: church of Saint Peter abbey (Erfurt, Germany)	Musée du Louvre (Paris, France), Inv. RF 1384	Tilman Riemenschneider or workshop. Supposed to have been commanded by the clergy for the church of Saint Peter abbey, Erfurt (Germany). Collection of a clergyman in Erfurt, the provost Würschmidt, in the 19th century. Sold after 1892 from a private collection in Dieburg near Frankfurt[18-22]. The attribution to Riemenschneider dates back to 1906[21]. Achieved by the Louvre Museum in 1904.	Fig.2S
Annunciation group: Virgin	~1485-1487	H 41, W 34, D 14	Unknown	Rijksmuseum (Amsterdam, Netherlands), Inv. BK-16986-A	Early alabaster work of Tilman Riemenschneider [19, 23]. By tradition from a monastery in Bamberg (Germany) [22], acquired by the Rijksmuseum in 1960.	Fig.2T
Annunciation group: Angel	~1485-1487	H 39.5, W 28.5, D 14	Unknown	Rijksmuseum (Amsterdam, Netherlands), Inv. BK-16986-A	Early alabaster work of Tilman Riemenschneider [19, 23]. By tradition from a monastery in Bamberg (Germany) [22], acquired by the Rijksmuseum in 1960.	Fig.2U
Saint Jerome with the lion	~1490-1495	H 37.8, W 28.1, D 15.9	Supposedly: church of Saint Peter abbey (Erfurt, Germany)	Cleveland Museum of Fine Art (Ohio, USA), Inv. CMA 1946.82	Tilman Riemenschneider or workshop. Together with the Louvre Annunciation, supposed to have been commanded by the clergy for the church of Saint Peter abbey,	Fig.2V

					Erfurt (Germany). Collection of a clergyman in Erfurt, the provost Würschmidt, in the 19th century. Both statues were sold after 1892 from a private collection in Dieburg near Frankfurt[18-20, 22]. Großmann attributed the Saint Jerome to Riemenschneider in 1909 [20]. Acquired by the Cleveland Museum of Fine Art in 1946.	
Anna Selbdritt	~1515-1520	H 36	Unknown	Museum für Franken (Würzburg, Germany), Inv. ZV67983	Late work of Tilman Riemenschneider or workshop. Devotional use. Acquired by the Museum für Franken in 2006[24]	Fig.2W
Annunciation	~1520-1525	H 32.2, W 22.0	Unknown	Daniel Katz Gallery Ltd. (London, UK)	Late work of Tilman Riemenschneider or workshop. Devotional use. Formerly in a private collection in Munich, exhibited by the Bayrisches Nationalmuseum, Munich, from 1998-2002[25]	Fig. 2X

S2 Table. Analyzed historical quarries Franconian alabaster deposits and their isotopic composition.

**Sample**	**Situation**	**Geology**	**$^{87}Sr/^{86}Sr$**	**2σ (m) $^{87}Sr/^{86}Sr$**	**$\delta^{34}S$ (‰ *vs.* V-CDT) ± 0.3 ‰**	**$\delta^{18}O$ (‰ vs. V-SMOW) ± 0.5 ‰**
Castell Schlossberg point 2	0.1 km S of Castell (Bavaria, Germany), vineyards	Upper Triassic, Ladinian to Karnian, local stratigraphy: Middle Keuper, uppermost part of the Myophoria beds, small nodules of slightly rose homogeneous gypsum in black marls.	0.708756	0.000009	14.8	13.5
Castell Schlossberg point 3 (2)	0.1 km S of Castell (Bavaria, Germany), vineyards	Upper Triassic, Ladinian to Karnian, local stratigraphy: Middle Keuper, upper part of the Myophoria beds, stratified greyish to white banked gypsum in black marls.	0.708535	0.000008	15.2	13.3
Castell Schlossberg point 5	0.8 km SW of Castell (Bavaria, Germany), limit vineyards-forest	Upper Triassic, Ladinian to Karnian, local stratigraphy: Middle Keuper, Estheria beds, massive greyish to white gypsum outcrop	0.708510	0.000008	14.9	13.9
Markt Seinsheim Point 1 (2)	1.5 km WSW Seinsheim (Bavaria, Germany), abandoned quarry of massive gypsum ("Grundgips"), some nodules.	Upper Triassic, Karnian, local stratigraphy: Middle Keuper, Myophoria beds, Grundgips gypsum layer	0.708599	0.000006	15.2	14.1
Markt Seinsheim Point 2 (1)	5.4 km SW Seinsheim (Bavaria, Germany), outcrop of massive to nodular gypsum, alabaster quality.	Upper Triassic, Ladinian to Karnian, local stratigraphy: Middle Keuper, Estheria beds	0.708494	0.000008	15.1	13.8
Markt Seinsheim Point 2 (4)	5.4 km SW Seinsheim (Bavaria, Germany), outcrop of massive to	Upper Triassic, Ladinian to Karnian, local stratigraphy: Middle Keuper, Estheria beds	0.708595	0.000010	15,1	13.6

	nodular gypsum, alabaster quality.					
Ickelheim Point 1 (2A)	2 km W Ickelheim (near Bad Windsheim, Bavaria, Germany), alabaster nodule, vineyards	Upper Triassic, Ladinian to Karnian, local stratigraphy: Middle Keuper, uppermost part of the Myophoria beds, nodular gypsum in black marls.	0.708649	0.000006	15.0	13.5
Ickelheim Point 3	2 km W Ickelheim (near Bad Windsheim, Bavaria, Germany), alabaster nodule, vineyards	Upper Triassic, Ladinian to Karnian, local stratigraphy: Middle Keuper, limit Myophoria beds-Estheria beds, nodular gypsum in black marls.	0.708709	0.000006	14.6	13.3
Ickelheim Point 4 "Neuer Bruch"	1.4 km WSW Ickelheim (near Bad Windsheim, Bavaria, Germany), alabaster nodule, marlstone outcrop identified as the “new quarry” mentioned by Hofmann 1757 [26]	Upper Triassic, Ladinian to Karnian, local stratigraphy: Middle Keuper, upper part of the Myophoria beds, nodular gypsum in black marls.	0.708356	0.000007	15.7	13.3
Ickelheim Point 5 (1) "Alter Bruch"	1.4 km SSW Ickelheim (near Bad Windsheim, Bavaria, Germany), alabaster fragment, weathered marlstone outcrop identified as the “old quarry” mentioned by Hofmann 1757 [26]	Upper Triassic, Ladinian to Karnian, local stratigraphy: Middle Keuper, upper part of the Myophoria beds, fragments of nodular gypsum in black marls.	0.708426	0.000007	15.2	12.6

S3 Table. Isotope signatures of artwork

Artwork	Current situation	$^{87}Sr/^{86}Sr$	2σ (m) $^{87}Sr/^{86}Sr$	$\delta^{34}S$ (‰ *vs.* V-CDT) ± 0.3 ‰	$\delta^{18}O$ (‰ vs. V-SMOW) ± 0.5 ‰
Swoon of the Virgin group	Liebieghaus Skulpturensammlung, (Frankfurt, Germany) Inv. 402	0.708603	0.000007	14.7	12.4
Swoon of the Virgin	Musée du Louvre (Paris, France), Inv. RF 1639	0.708589	0.000007	14.4	13.2
"Three Maries" (Swoon of the Virgin)	National Museum, (Warsaw, Poland), Inv. Śr.402	0.708616	0.000008	14.6	14.6
Pietà	Mittelrheinmuseum (Koblenz, Germany), Inv. P 1990/13	0.708527	0.000007	14.8	14.1
Pietà	Rijksmuseum (Amsterdam, Netherlands), Inv. BK-NM-11667	0.708635	0.000007	14.5	12.3
Pietà	Museum am Dom (Würzburg, Germany), Inv. 491	0,708605	0,000007	14,7	13
Pietà	Deutschordensmuseum (Bad Mergentheim, Germany)	0,708644	0,000007	14,7	12,6
Apostle Saint Paul	Liebieghaus Skulpturensammlung, (Frankfurt, Germany) Inv. 406	0.708658	0.000006	14.5	12.3
Apostle	Liebieghaus Skulpturensammlung, (Frankfurt, Germany) Inv. 409	0.708624	0.000007	14.6	11.3
Apostle Saint Andrew	Liebieghaus Skulpturensammlung, (Frankfurt, Germany) Inv. 417	0.708640	0.000006	14.5	12.4
Apostle Saint Peter (replaced head)	Liebieghaus Skulpturensammlung, (Frankfurt, Germany) Inv. 418	0.707111	0.000008	11.8	11.4
Apostle	Musée du Louvre (Paris, France) Inv. RF 4402	0.708630	0.000007	14.5	13.0
Apostle	Musée de l'Hôtel Sandelin (Saint-Omer, France), Inv. 2911.3	0.708675	0.000008	14.6	12.3
Apostle	Musée de l'Hôtel Sandelin (Saint-Omer, France) Inv. 2911.4	0.708638	0.000009	14.6	12.5
Apostle Saint Philip	J.P. Getty Museum (Los Angeles, USA), Inv. 2015.58	0.708648	0.000006	14.5	12.1
Apostle	Saint Victor Church (Schwerte, Germany)	0.708622	0.000008	14.5	13.1
Apostle (pedestal)	Saint Victor Church (Schwerte, Germany)	0.708642	0.000008	14.3	13.1
Annunciation group: Virgin	Musée du Louvre (Paris, France), Inv. RF 1384	0.708743	0.000009	14.3	13.6
Annunciation group: Virgin	Rijksmuseum (Amsterdam, Netherlands), Inv. BK-16986-A	0.708647	0.000007	14.5	12.4
Annunciation group: Angel	Rijksmuseum (Amsterdam, Netherlands), Inv. BK-16986-A	0.708680	0.000010	14.5	13.2

Saint Jerome with the lion	Cleveland Museum of Fine Art (Ohio, USA), Inv. CMA 1946.82	0.708608	0.000009	14.4	11.8
Anna Selbdritt	Museum für Franken (Würzburg, Germany), Inv. ZV67983	0.708728	0.000008	14.5	11.9
Annunciation	Daniel Katz Gallery Ldt. (London, UK)	0,708670	0,000006	14,6	13,9

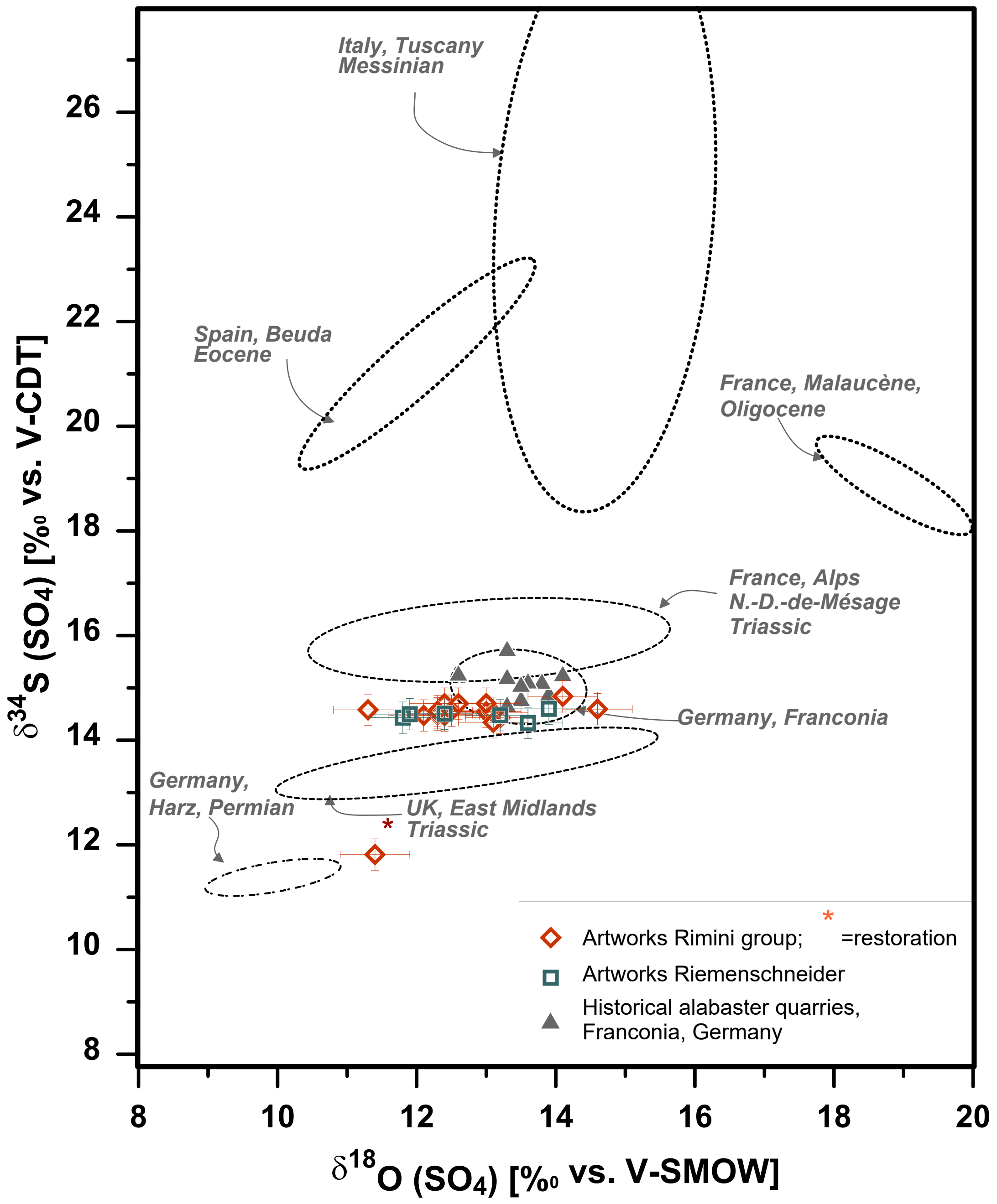

S4 Fig. Isotope fingerprints of sulphur vs. oxygen (δ^{34}S *vs.* δ^{18}O) of the artwork attributed to the workshops of the Rimini Master and Tilman Riemenschneider and of the alabaster quarries in Franconia (Germany). For comparison: principal deposits previously identified to have delivered alabaster for 14th to 16th century sculpture in W Europe [27-29], δ^{34}S renormalised to V-CDT.

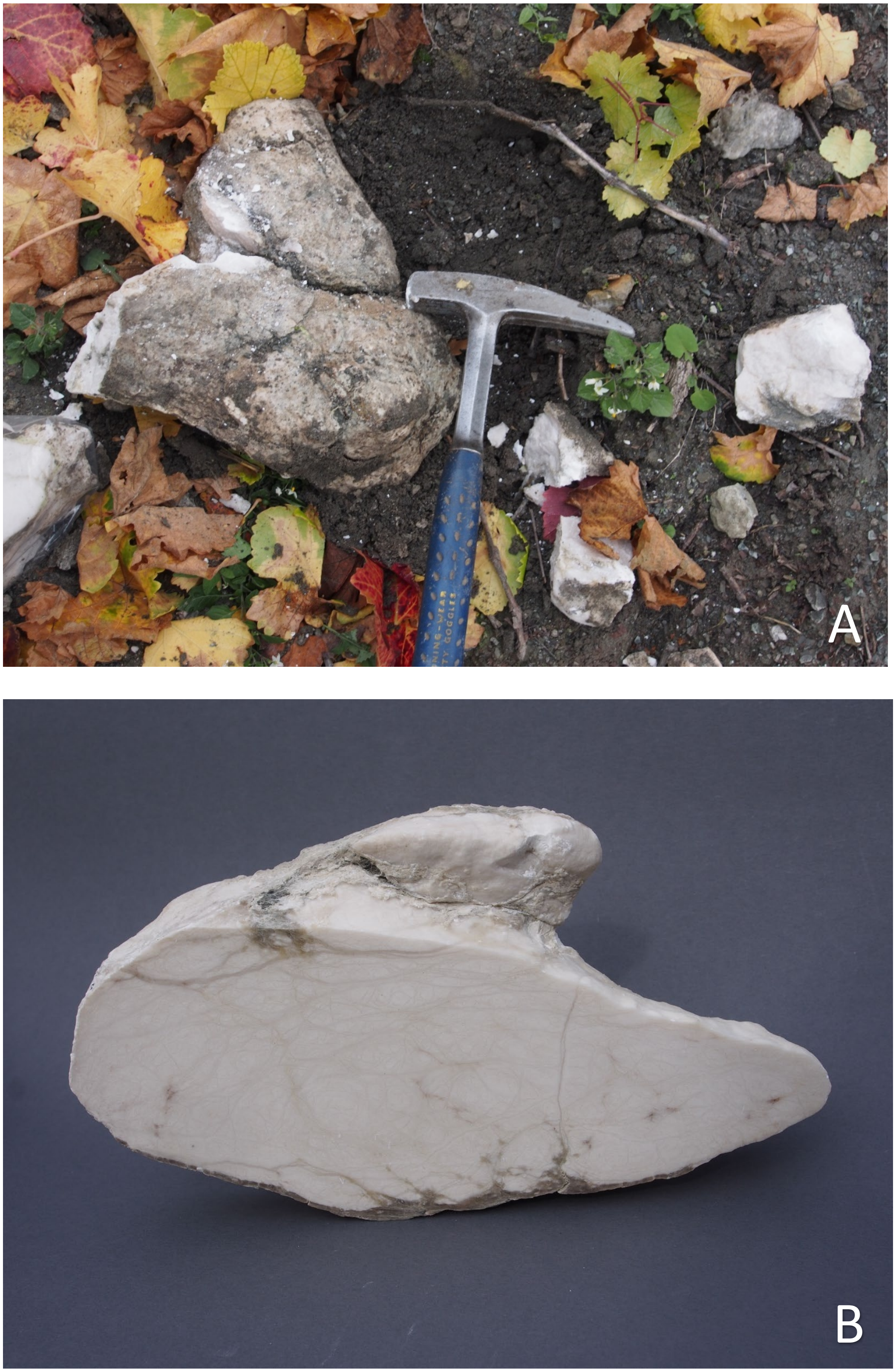

S5 Fig. Alabaster nodules from the Ickelheim deposit. (A) marlstone outcrop with decimetric alabaster nodule in the vineyards (B) cut and polished alabaster nodule, largest diameter: 21 cm
